# Supplementary material for: A dynamic foot model for predictive simulations of human gait reveals causal relations between foot structure and whole-body mechanics
Source: PLoS Comput Biol. 2024 Jun 20;20(6):e1012219. doi: 10.1371/journal.pcbi.1012219 (PMC11218950; doi:10.1371/journal.pcbi.1012219)
Supplement: S1 Text — Contains Figs A to AO and Tables A to O with their individual captions. (PDF) [file pcbi.1012219.s001.pdf]

A dynamic foot model for predictive simulations of human gait reveals causal relations between foot structure and whole-body mechanics - Supplementary material

Lars D'Hondt[1]*, Friedl De Groote[1], and Maarten Afschrift[2]

[1]Department of Movement Sciences, KU Leuven, Leuven, Belgium.

[2]Department of Human Movement Sciences, Vrije Universiteit, Amsterdam, The Netherlands.

*Corresponding author: lars.dhondt@kuleuven.be

Table of Contents

1. Properties of muscles spanning the ankle 3
   Achilles tendon stiffness 4
   Passive isometric ankle moment 6
   Triceps surae maximal isometric force 8
2. Midtarsal and MTP joint axis orientation 10
3. Midtarsal joint stiffness due to ligaments 12
4. Plantar fascia stiffness 13
5. Plantar intrinsic muscle parameter sensitivity 15
   Maximal isometric force 15
   Optimal fibre length 16
   Tendon slack length 17
6. Foot-ground contact 18
   Stiffness 18
   Position heel sphere 20
   Configuration 22
7. Contributions of extrinsic foot muscles 24
8. Approximating conditional statements with a hyperbolic tangent 26
9. Convergence analysis 27
10. Marker protocol 28
11. Predictive gait simulations with 3-segment and 4-segment foot models 29
   Cross-correlation coefficients 29
   Weighted root mean square errors 32
   Additional Figs 35
12. Reducing the ability to stiffen the foot 39
13. Foot arch height 43
14. Plantar intrinsic muscle nerve block 44
References 47

1. Properties of muscles spanning the ankle

Table A Modelled muscle-tendon units of the foot and ankle

Muscle	Origin segment	Insertion segment
Medial gastrocnemius	femur	hindfoot
Lateral gastrocnemius	femur	hindfoot
Soleus	tibia	hindfoot
Tibialis anterior	tibia	midfoot-forefoot
Tibialis posterior	tibia	midfoot-forefoot
Peroneus longus	tibia	midfoot-forefoot
Peroneus brevis	tibia	midfoot-forefoot
Peroneus tertius	tibia	midfoot-forefoot
Extensor hallucis longus	tibia	toes
Extensor digitorum longus	tibia	toes
Flexor hallucis longus	tibia	toes
Flexor digitorum longus	tibia	toes
Plantar intrinsic muscle	hindfoot	toes

Achilles tendon stiffness

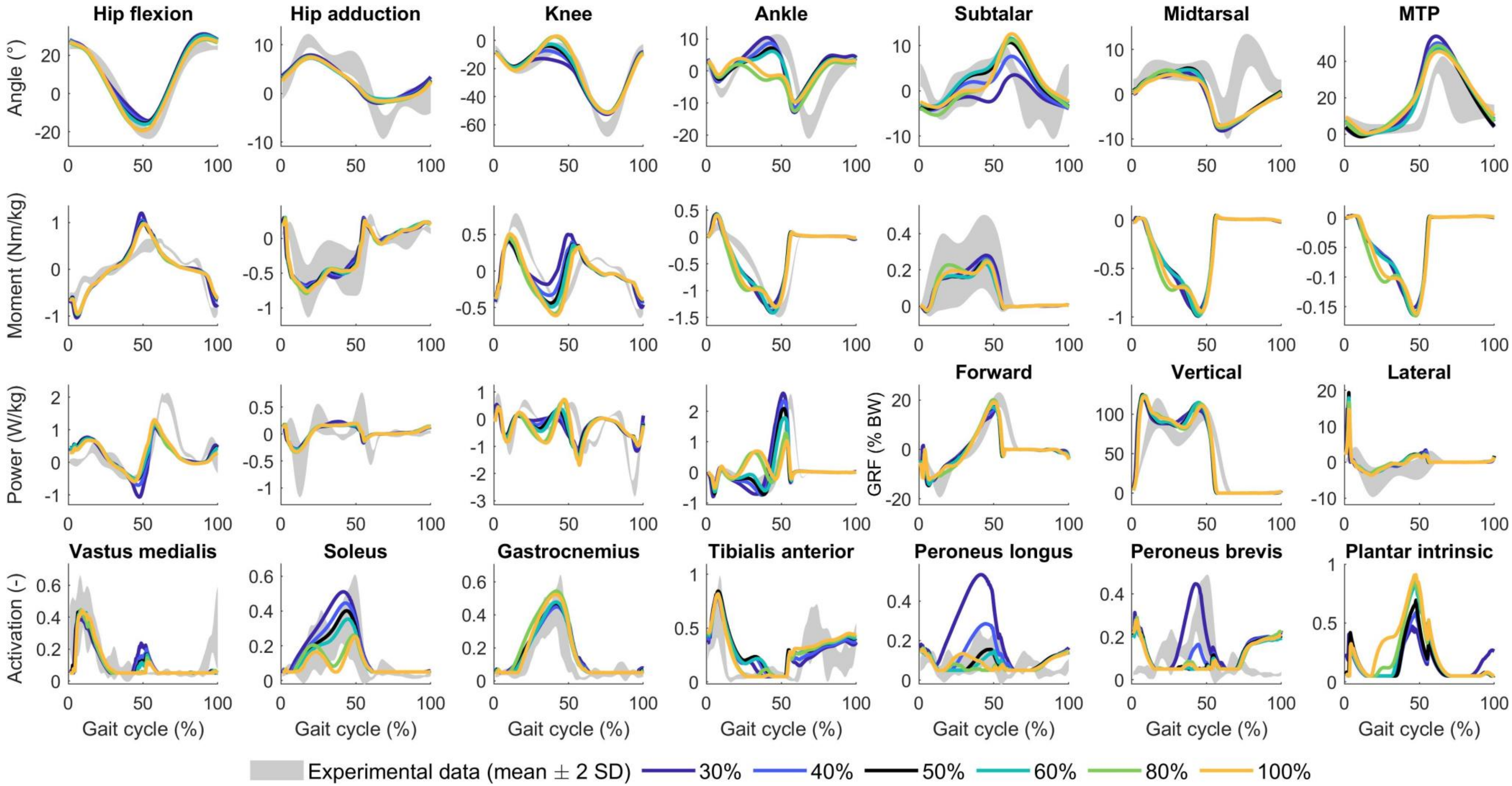

Fig A Effect of Achilles tendon stiffness for 4-segment foot model. Normalised stiffness is reduced to a percentage of its default value (1).

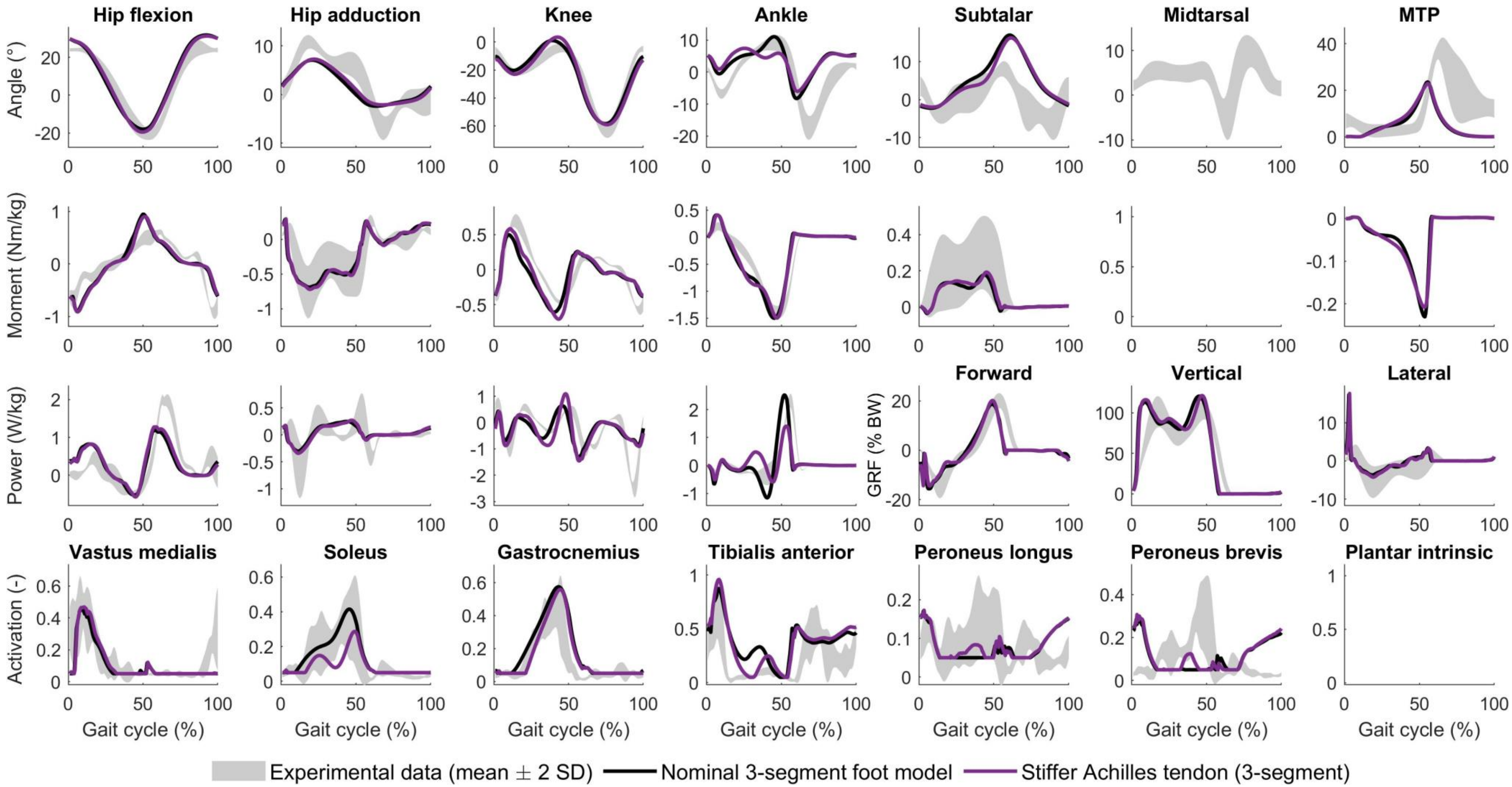

Fig B Effect of Achilles tendon stiffness for 3-segment foot model. Normalised stiffness is reduced to half its default value (1).

Passive isometric ankle moment

We adapted the parameters related to passive stiffness of the muscles spanning the ankle joint as the current passive ankle moment-angle relation does not match experimental data (2). Passive stiffness of all muscles crossing the ankle joint was increased by shifting their passive force-length characteristic (1) with 10% of the optimal fibre length towards smaller fibre lengths. The resulting passive ankle moments are more consistent with measurements (2) (Fig C).

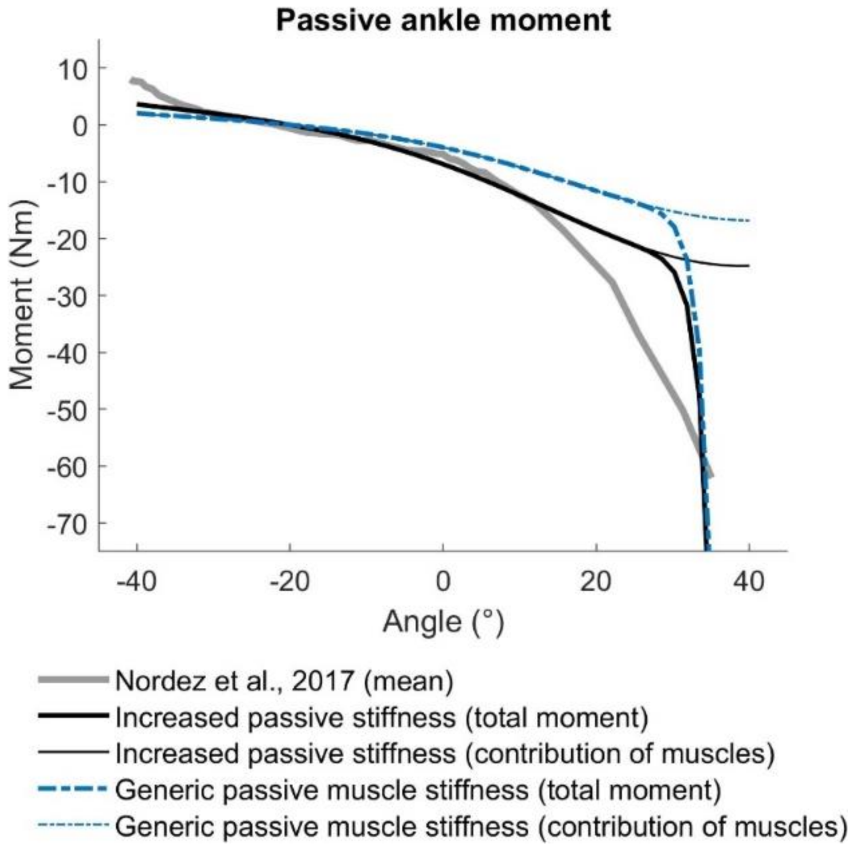

Fig C Passive isometric ankle moments. Dorsiflexion is positive. Thin lines show the contribution of muscles. Thick lines show total, also including coordinate limit torques (3). Reference data was digitised from Nordez et al (2). Increasing passive stiffness, by shifting the passive force-length characteristic of all muscles crossing the ankle towards 10% shorter normalised fibre lengths (1), results in more accurate passive moments with the range expected in walking (-20 – 15°).

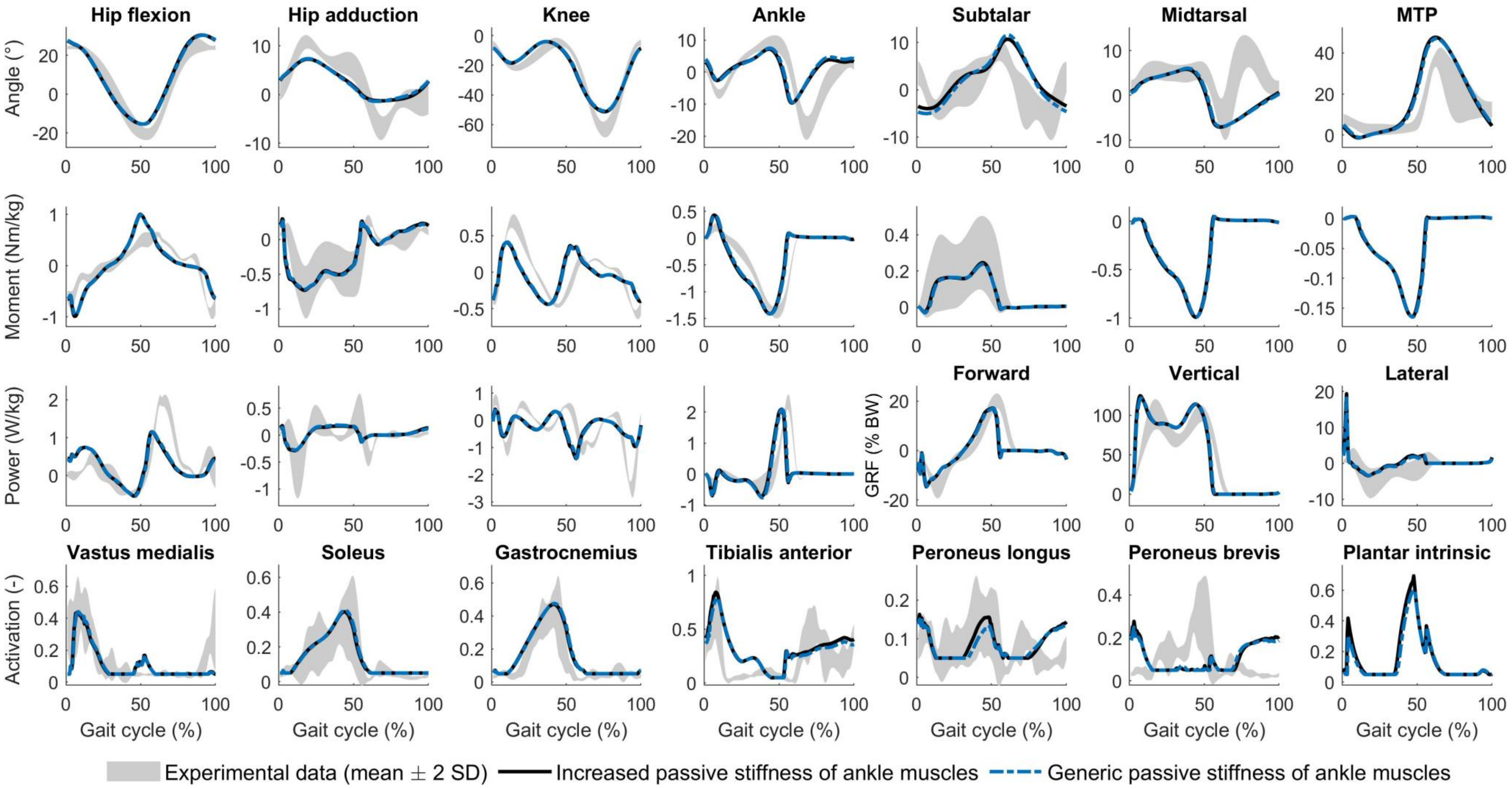

Fig D Effect of shifting the passive force-length characteristic of all muscles crossing the ankle towards 10% shorter normalised fibre lengths (1).

Triceps surae maximal isometric force

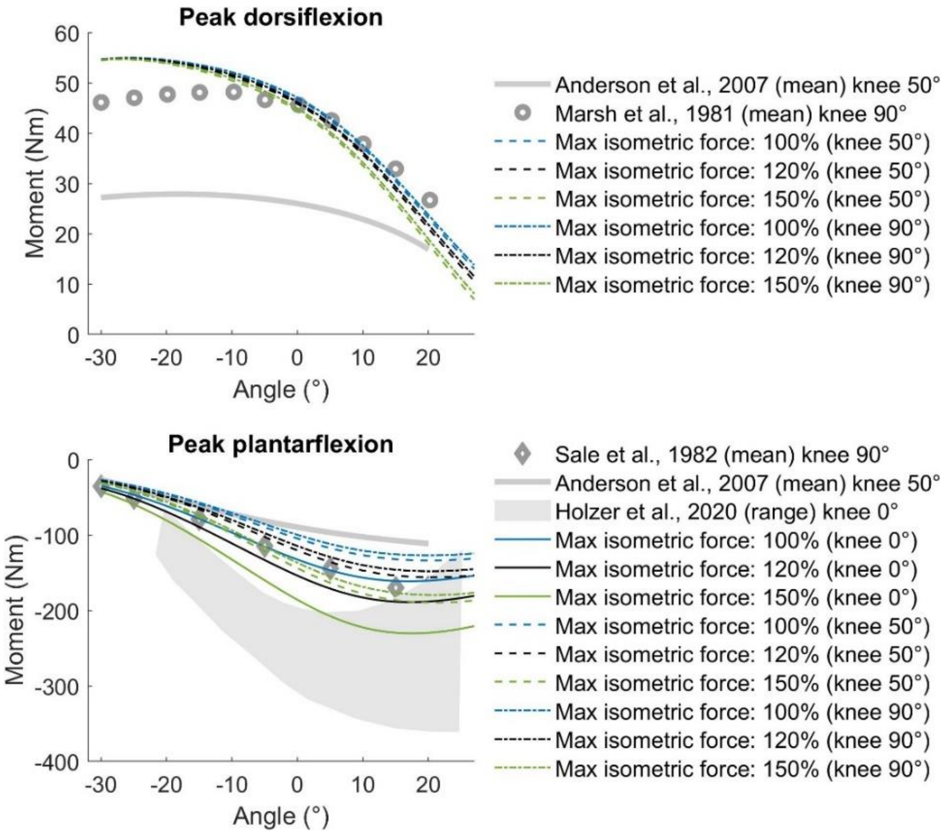

Fig E Effect of triceps surae maximal isometric force on maximal isometric ankle moments. Dorsiflexion is positive. Knee flexion angles were adjusted to match experimental conditions. We calculated peak isometric ankle moments by assuming maximal activation of agonists and 1% activation of antagonists. Experimental data taken from (4–7).

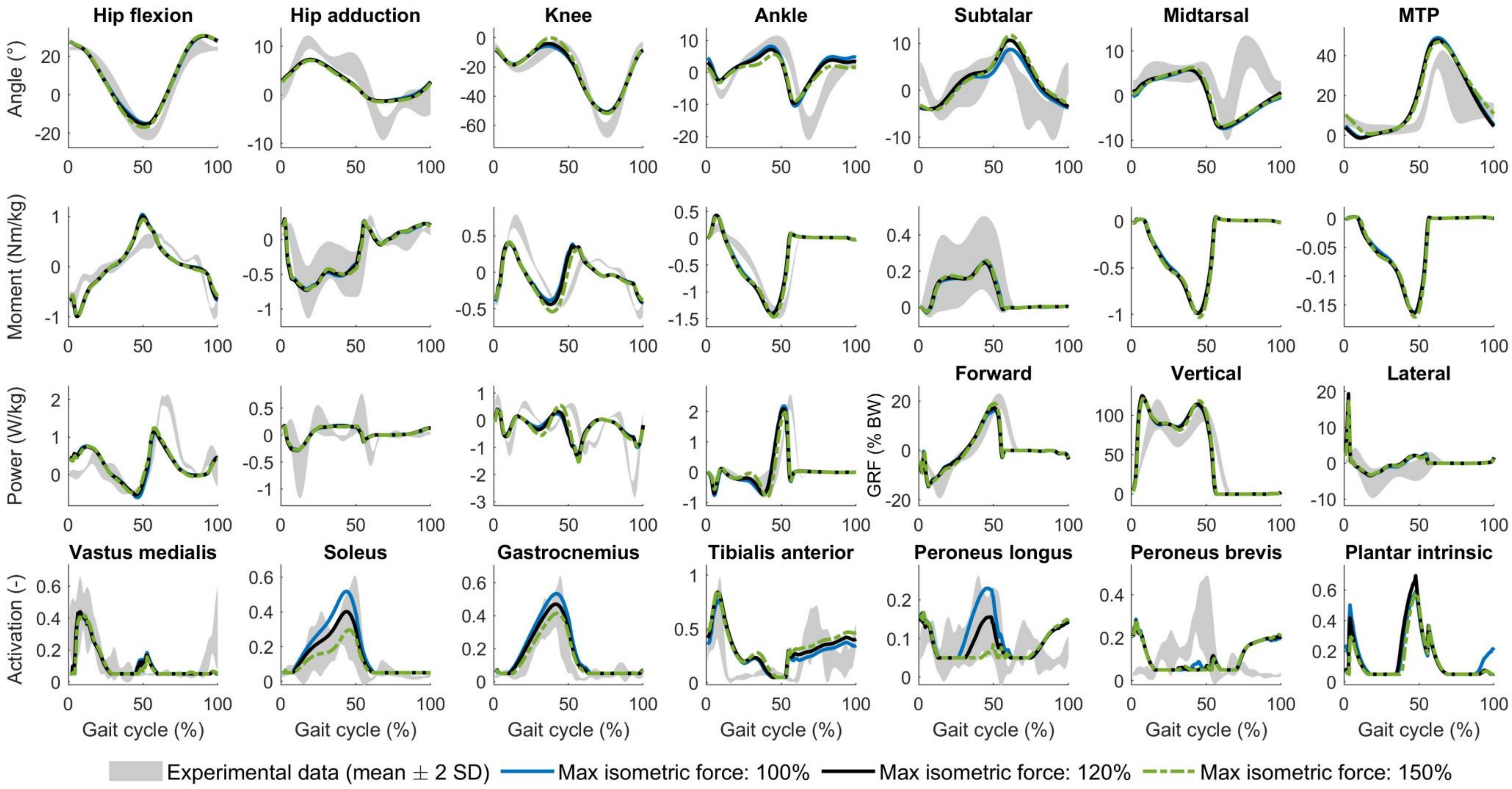

Fig F Effect of triceps surae maximal isometric force.

2. Midtarsal and MTP joint axis orientation

We changed the orientation of the metatarsophalangeal (MTP) joint axis to obtain more realistic behaviour of the flexor hallucis longus and extensor digitorum longus tendons. Assuming an MTP axis normal to the sagittal plane causes the moment arm of the flexor digitorum longus with respect to the MTP joint to decrease with increasing MTP extension. For extension of 30° and more, the moment arm is lower than 3 mm. This is inconsistent with the anatomy of the tendons passing over the metatarsal heads. We avoided this by modelling an MTP axis oriented according to the MTP plantarflexion-dorsiflexion axis proposed by Malaquias et al. (8). We also included a wrapping cylinder (radius 9.5 mm (9,10)), representing the first metatarsal head, to the path of the flexor hallucis longus.

We modelled the foot arch as a midtarsal joint with a single rotational degree of freedom, connecting calcaneus and midfoot segments. Midfoot and forefoot segments are rigidly connected. Midtarsal joint centre, segment definitions, and segment mass properties were taken from Malaquias et al. (8). We simulated walking for models with seven different midtarsal joint axis orientations (Fig G). The nominal orientation of the midtarsal joint axis is set according to the mean finite helical axis calculated from the motion capture data during the stance phase of walking using the algorithm provided by Ancillao (11). We tested an alternative axis oriented normal to the sagittal plane. Axis 1 and 5 are respectively the anterior-posterior and oblique axis defined by Malaquias et al. (8). Axis 2, 3, and 4 are interpolations at 30° increments.

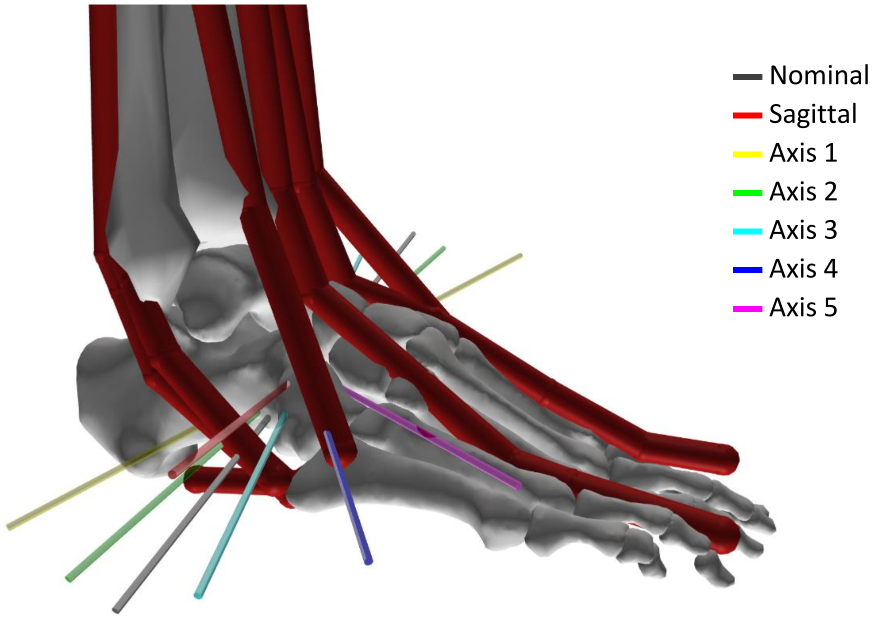

Fig G Orientation of the different midtarsal joint axes considered. Visualised via OpenSim (12,13).

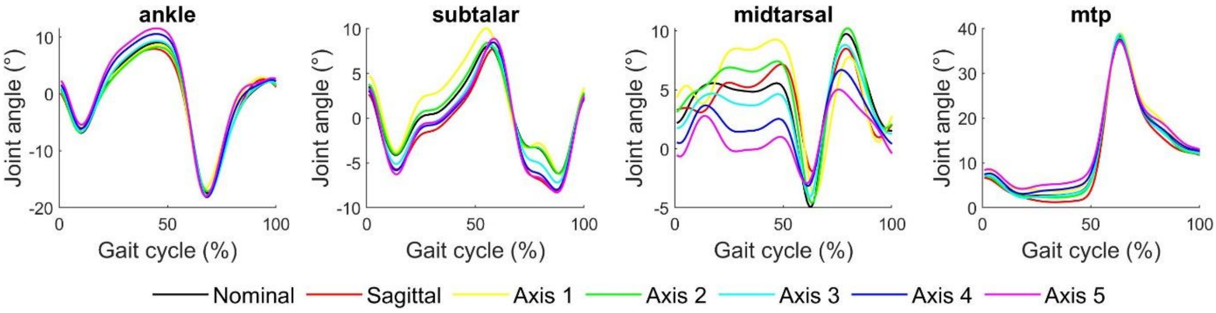

Fig H Effect of midtarsal joint axis orientation on inverse kinematics. Mean inverse kinematics of 10 strides overground walking at self-selected speed (1.33 m s^{-1}). Proximal joints are not shown because there was no considerable effect.

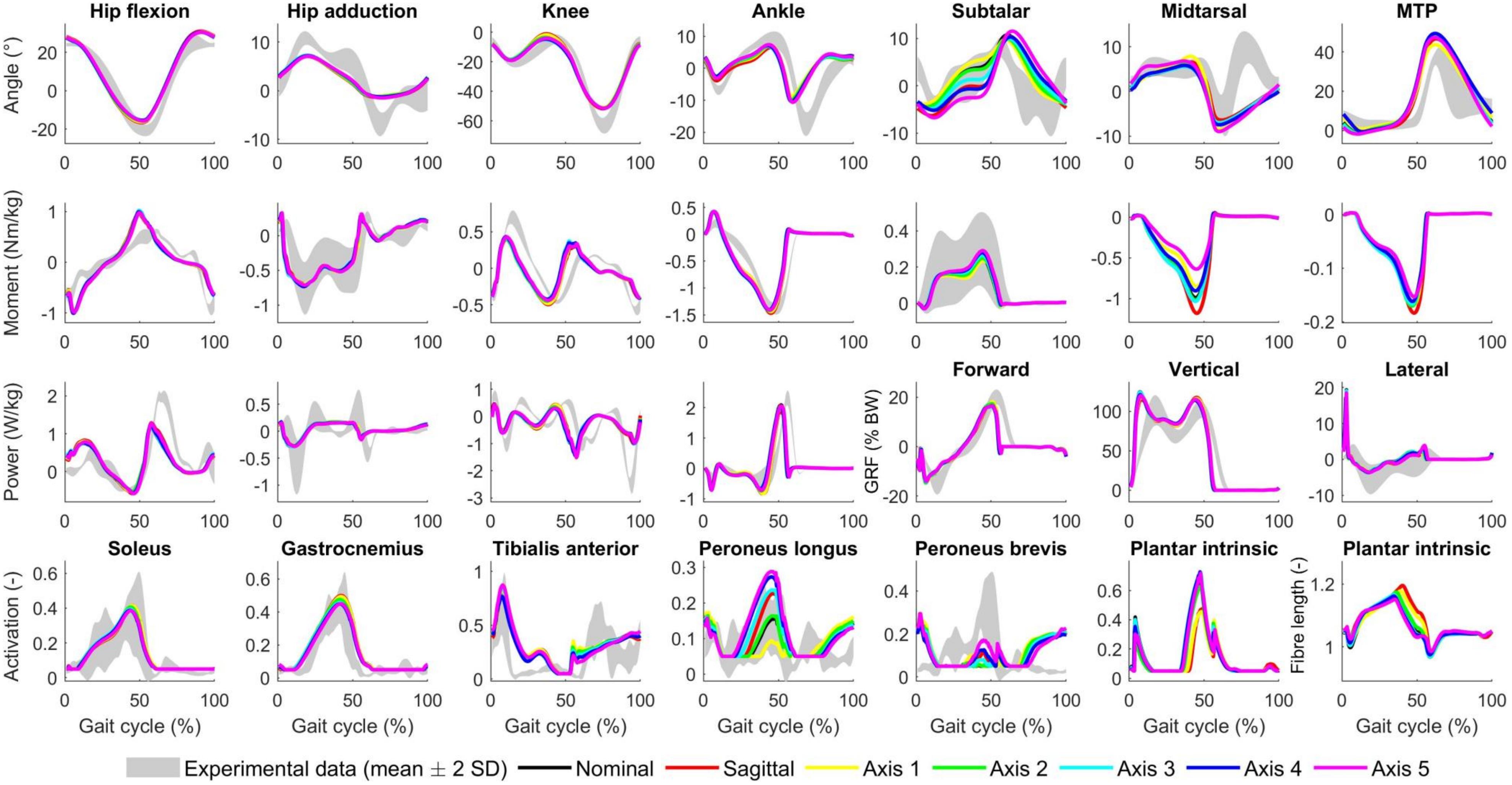

Fig I Effect of midtarsal joint axis orientation on simulated gait.

3. Midtarsal joint stiffness due to ligaments

We computed the rotational stiffness of the midtarsal joint by combining the contributions of individual ligaments. We included long plantar ligaments, calcaneo-navicular (plantar and bifurcate) ligaments, and calcaneo-cuboid (plantar, dorsal, and bifurcate) ligaments as modelled by Malaquias et al. (8). We used a stiffening stress-strain characteristic (14) but replaced the polynomial function by an exponential function that closely fitted the original polynomial function to improve computational stability (Fig J).

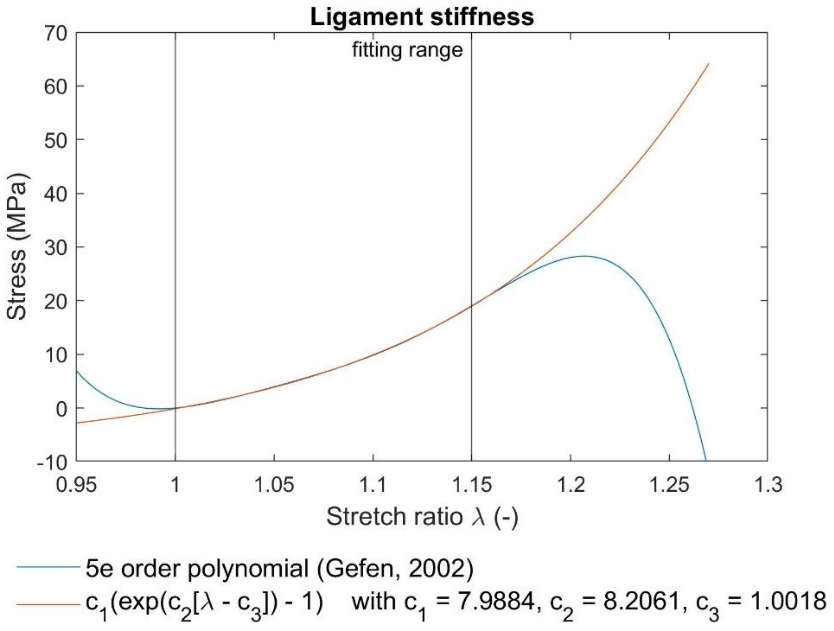

Fig J Exponential description of ligament stiffness characteristic from (14). We fit an exponential expression, because extrapolating the polynomial is numerically unstable.

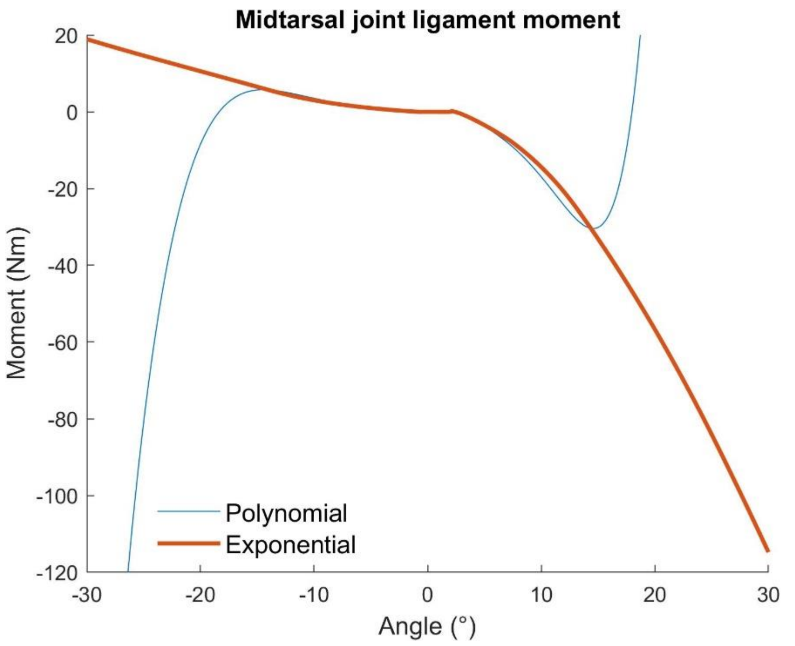

Fig K Total angle-moment characteristic of ligaments spanning the midtarsal joint.

4. Plantar fascia stiffness

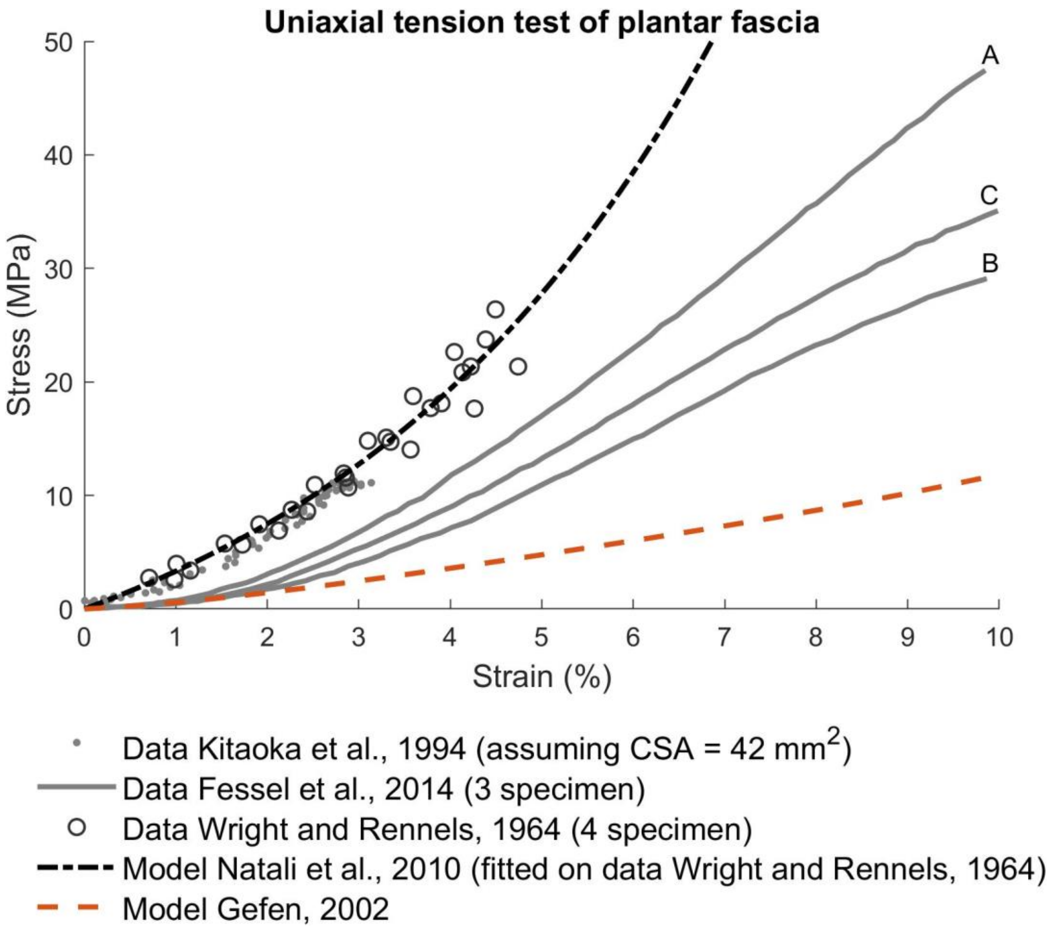

Fig L Plantar fascia stress-strain. Experimental data digitised from Kitaoka et al. (15), Fessel at al. (16), and Wright and Rennels (17). Since Kitaoka et al. report force, and no cross-sectional area, we calculated the stress assuming a cross-sectional area of 42 mm². This value is based on measurements of cadavers of similar ages (15,16,18). Plantar fascia models taken from Natali et al. (19), and Gefen (14).

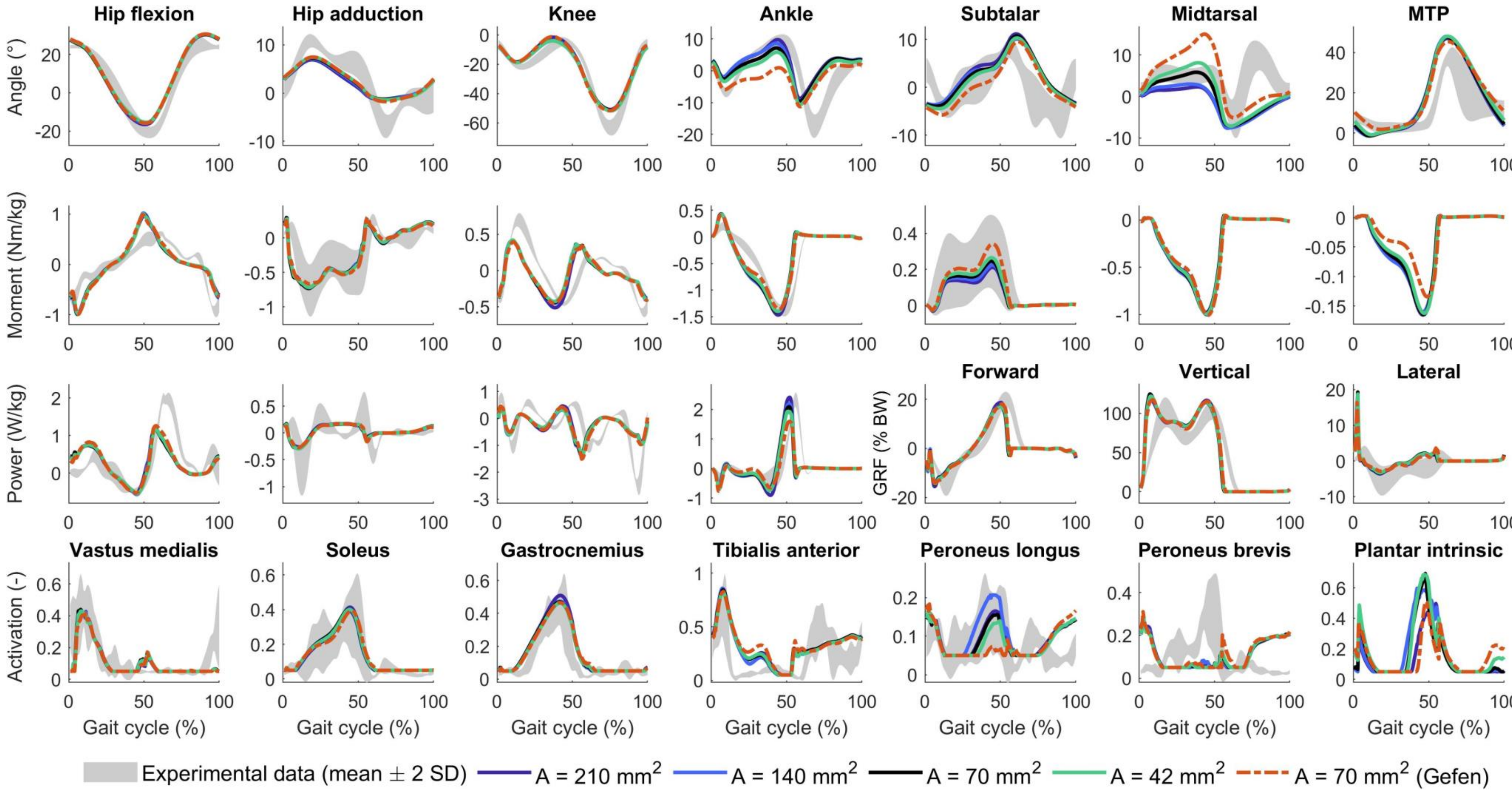

Fig M Effect of plantar fascia stiffness on simulated gait. Full lines show the results of combining the stress-strain characteristic from Natali et al. (19) with different cross-sectional areas. Dashed lines show the result of the stress-strain characteristic from Gefen (14) and a cross-sectional area of 70 mm².

5. Plantar intrinsic muscle parameter sensitivity

Maximal isometric force

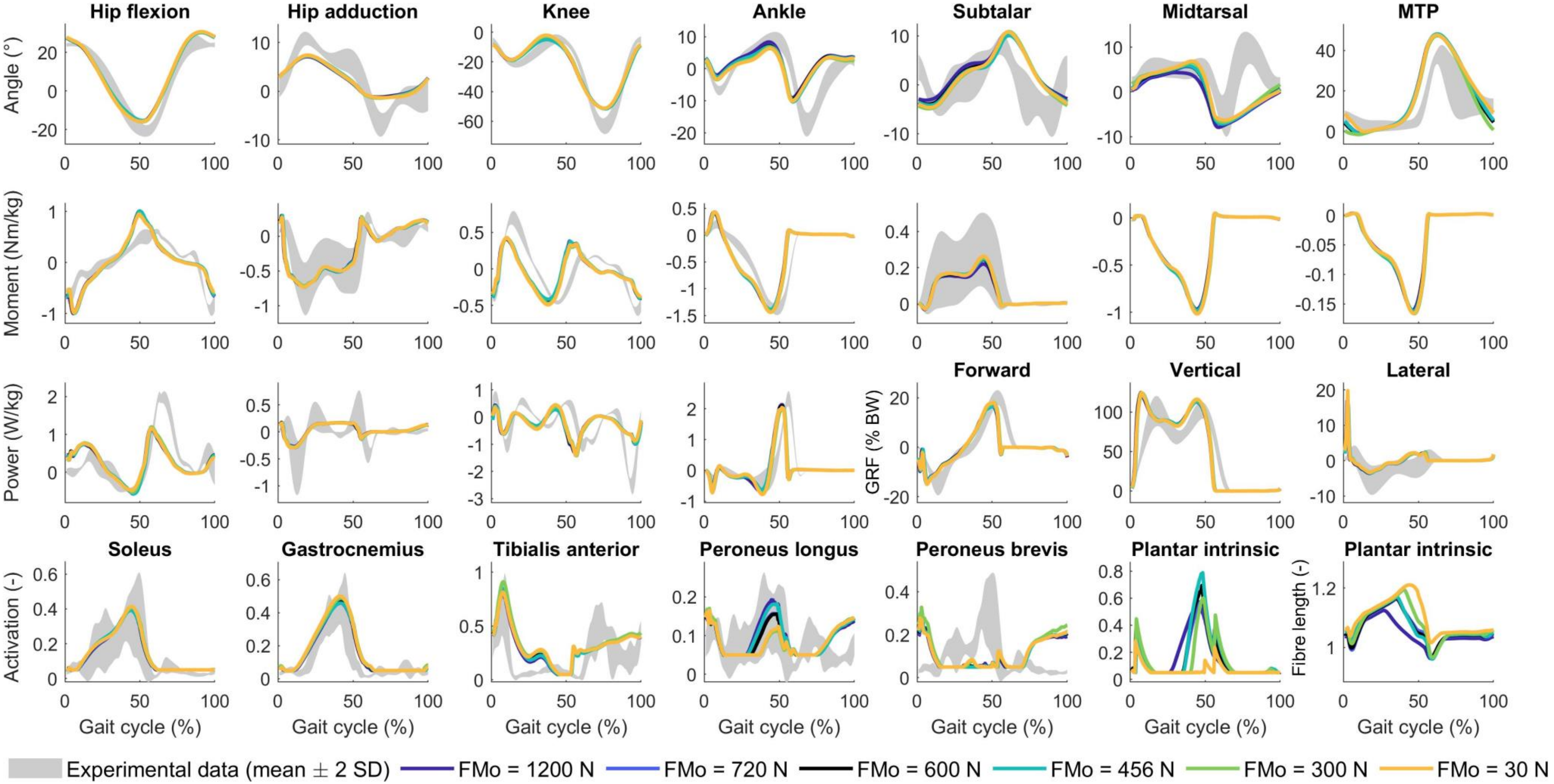

Fig N Effect of plantar intrinsic muscle maximal isometric force (FMo) on simulated gait.

Optimal fibre length

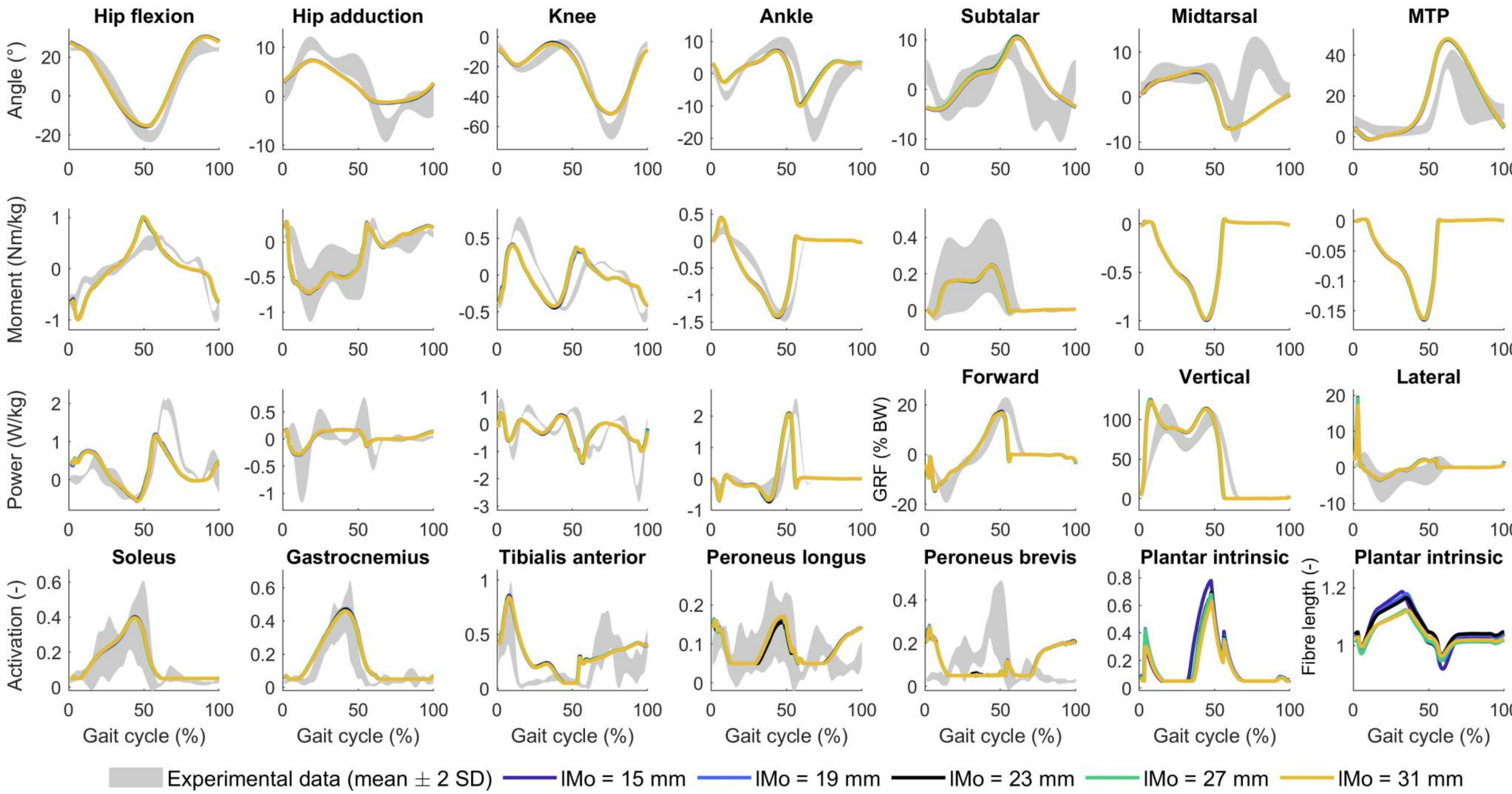

Fig O Effect of plantar intrinsic muscle optimal fibre length (lMo) on simulated gait.

Tendon slack length

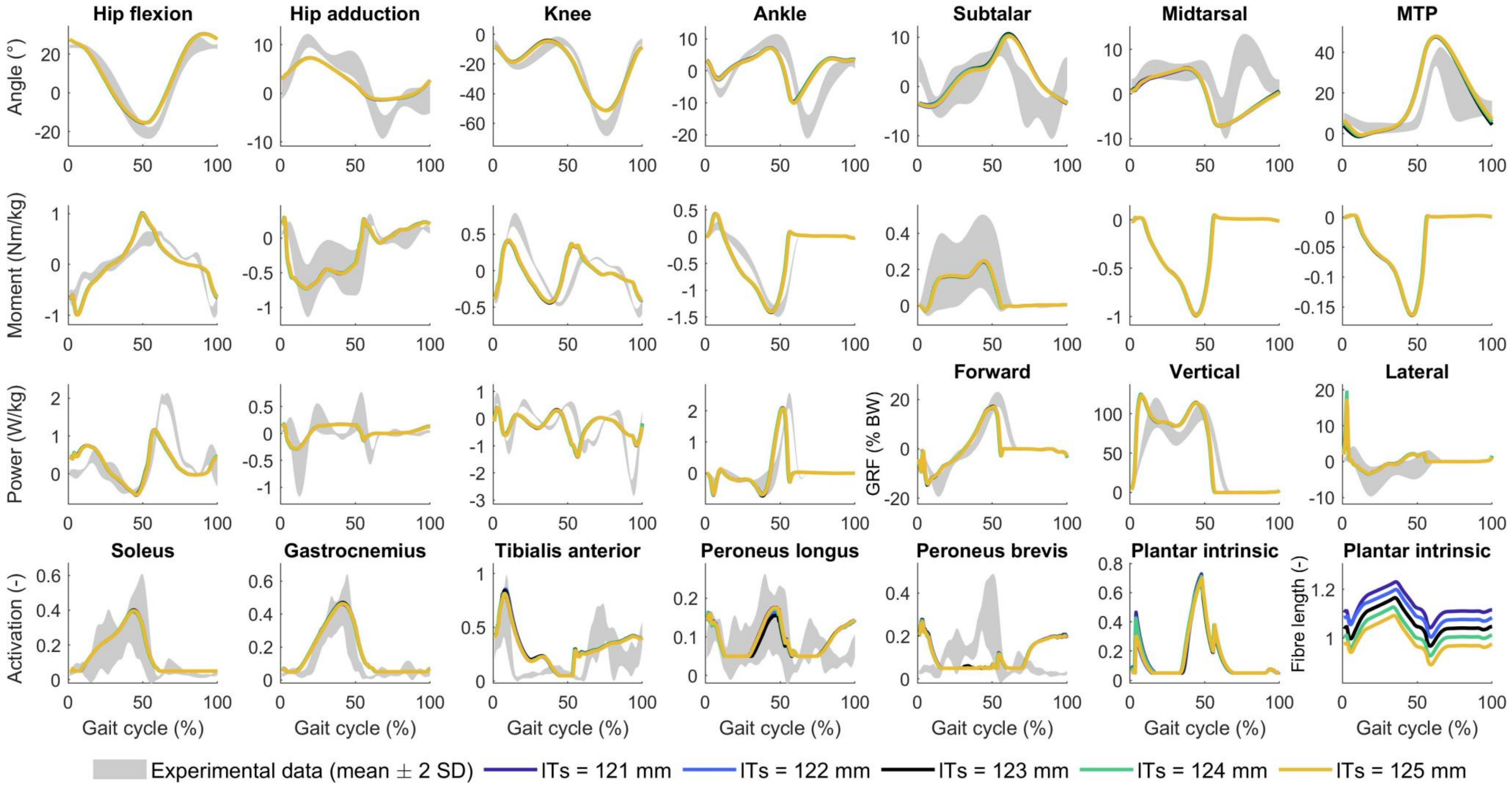

Fig P Effect of plantar intrinsic muscle tendon slack length (lTs) on simulated gait.

6. Foot-ground contact

Stiffness

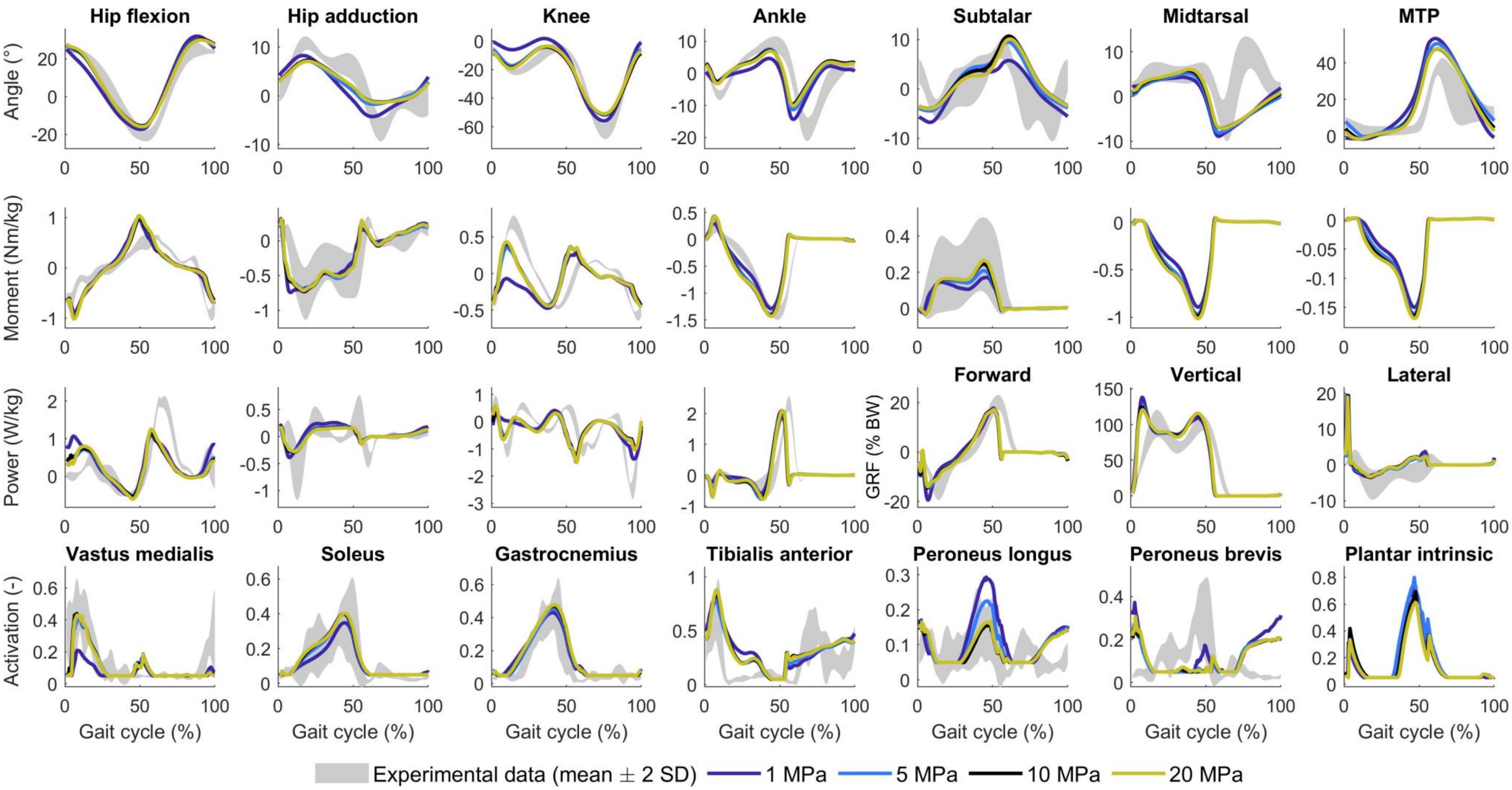

Fig Q Effect of foot-ground contact stiffness (20) on simulated gait for the 4-segment foot model.

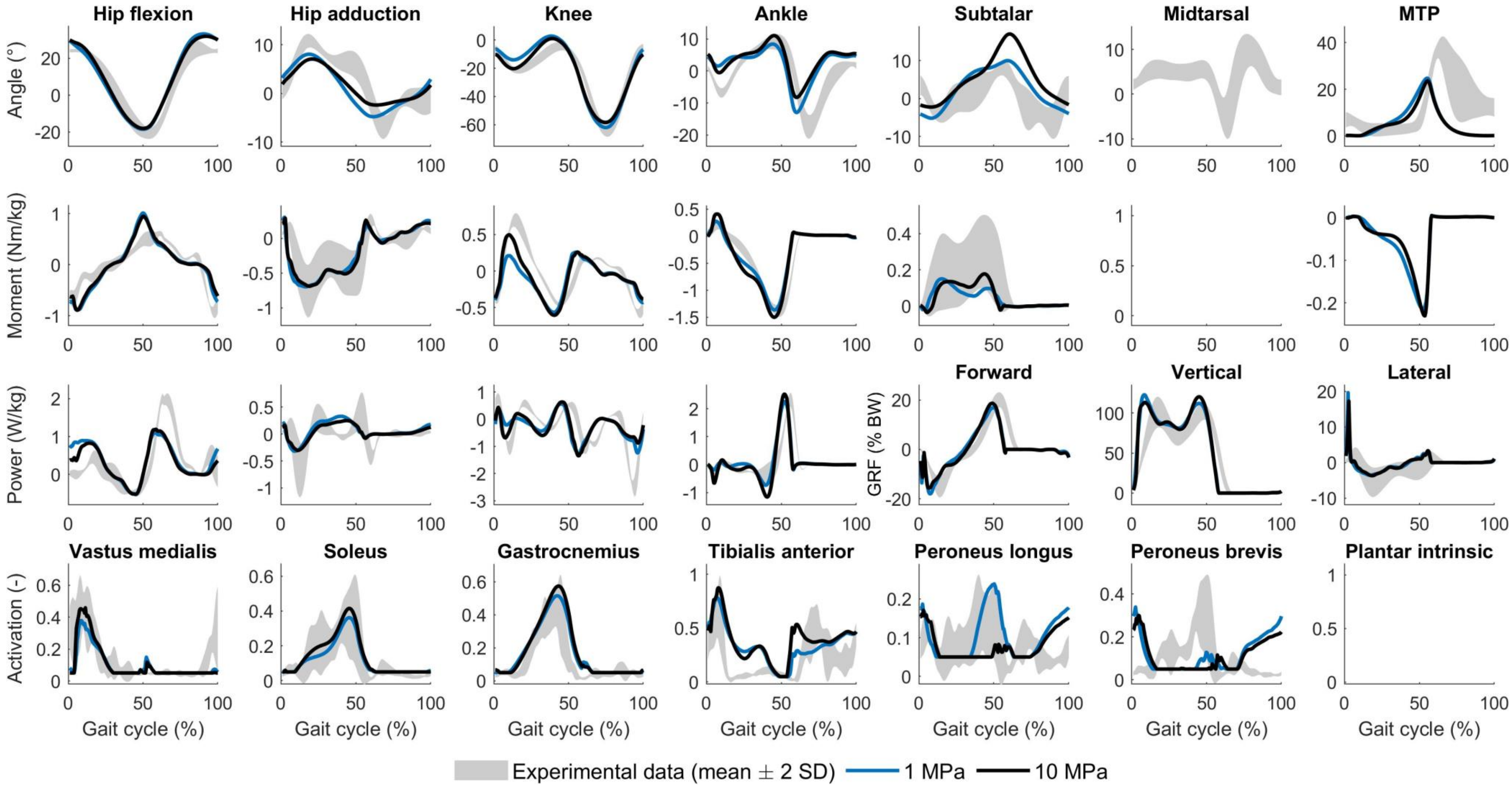

Fig R Effect of foot-ground contact stiffness (20) on simulated gait for the 3-segment foot model.

Position heel sphere

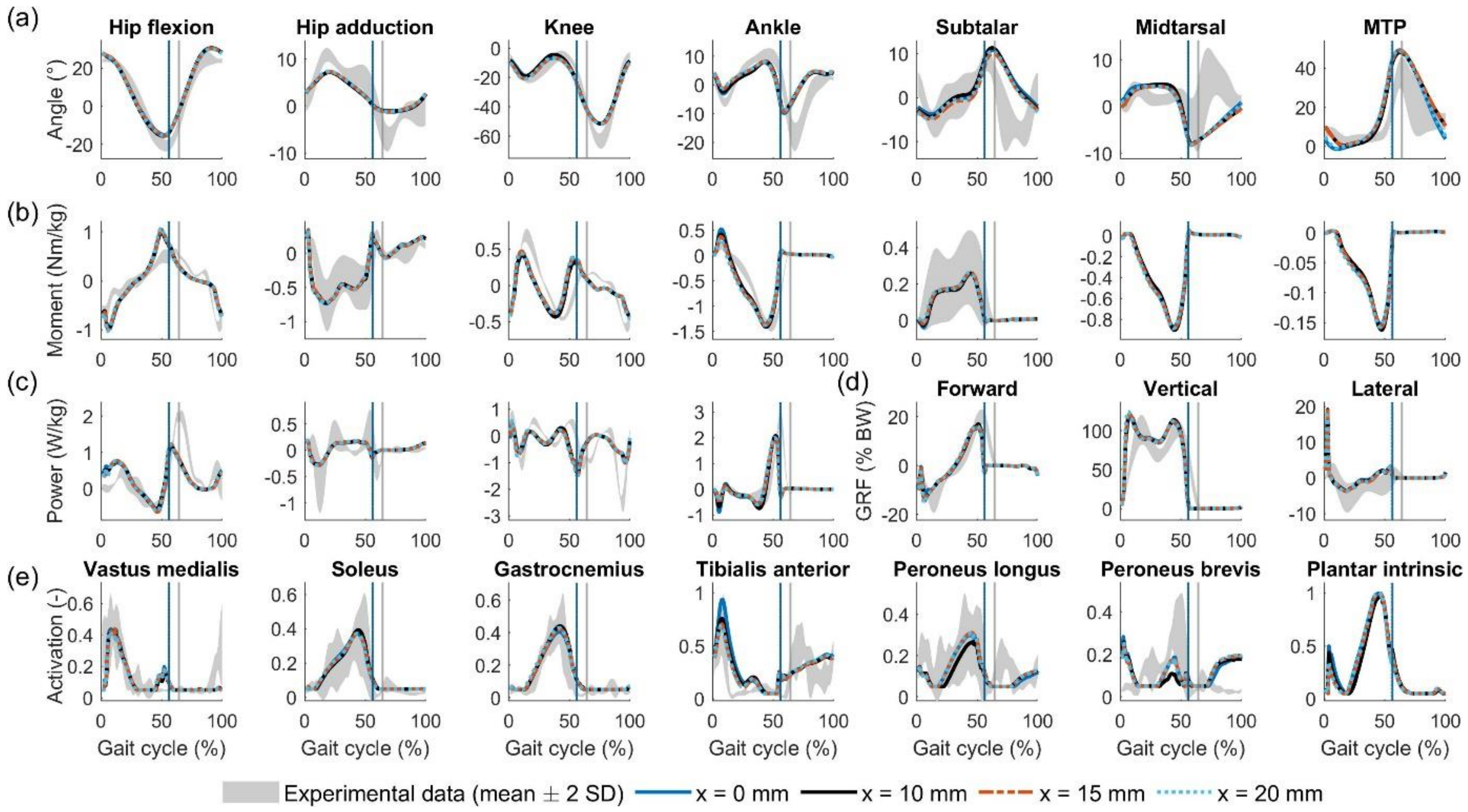

Fig S Effect of heel contact sphere position on simulated gait with 4-segment foot model. The nominal models consider the sphere centre at x = 10 mm. Placing the heel contact further back (lower x) results in increased tibialis anterior activation in early stance. This can be attributed to the GRF having a larger lever around the ankle, thus requiring more muscle force to counter.

(a) Kinematics. (b) Kinetics. (c) Joint powers. (d) Ground reaction forces, expressed as % body weight. (e) Muscle activation. Gastrocnemius indicates the medial gastrocnemius.

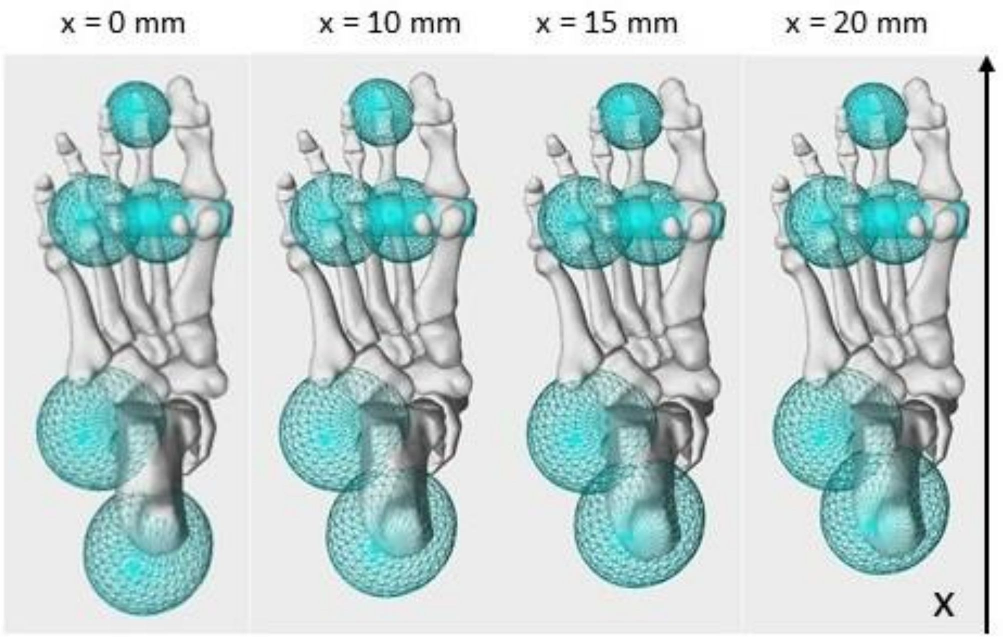

Fig T X-position of the heel contact sphere used for simulations in Fig S. The nominal models consider the sphere centre at x = 10 mm. Visualised via OpenSim (12,13).

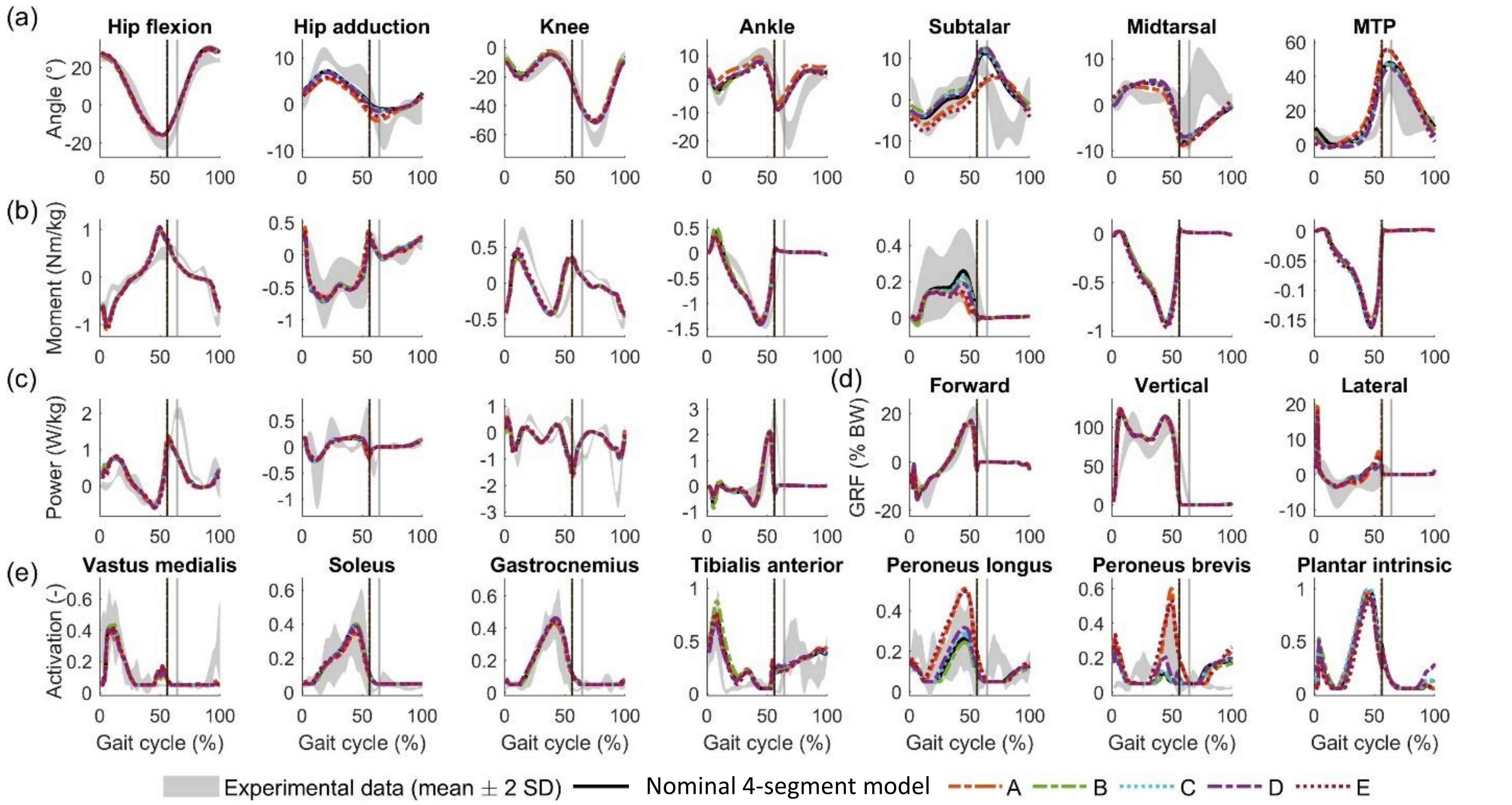

Fig U Effect of contact sphere configuration on simulated gait with 4-segment foot model. Only subtalar joint and peroneus muscles are sensitive. (a) Kinematics. (b) Kinetics. (c) Joint powers. (d) Ground reaction forces, expressed as % body weight. (e) Muscle activation. Gastrocnemius indicates the medial gastrocnemius.

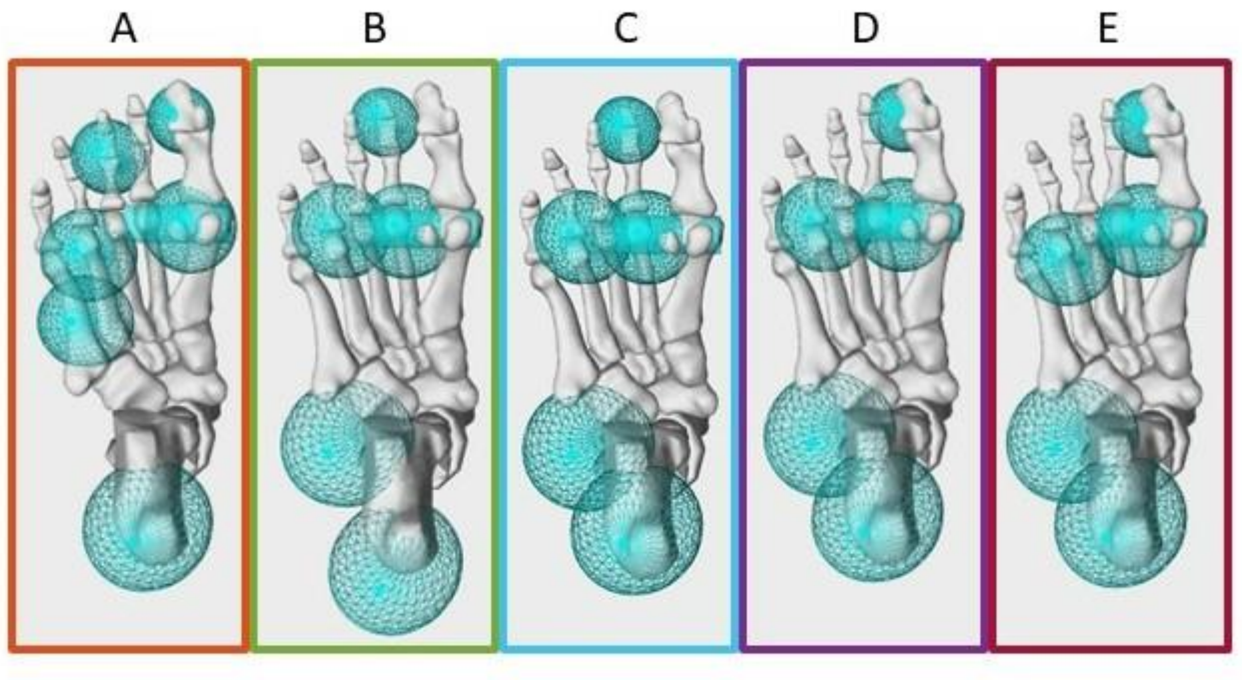

Fig V Contact sphere configurations used for simulations in Fig U. Visualised via OpenSim (12,13).

7. Contributions of extrinsic foot muscles

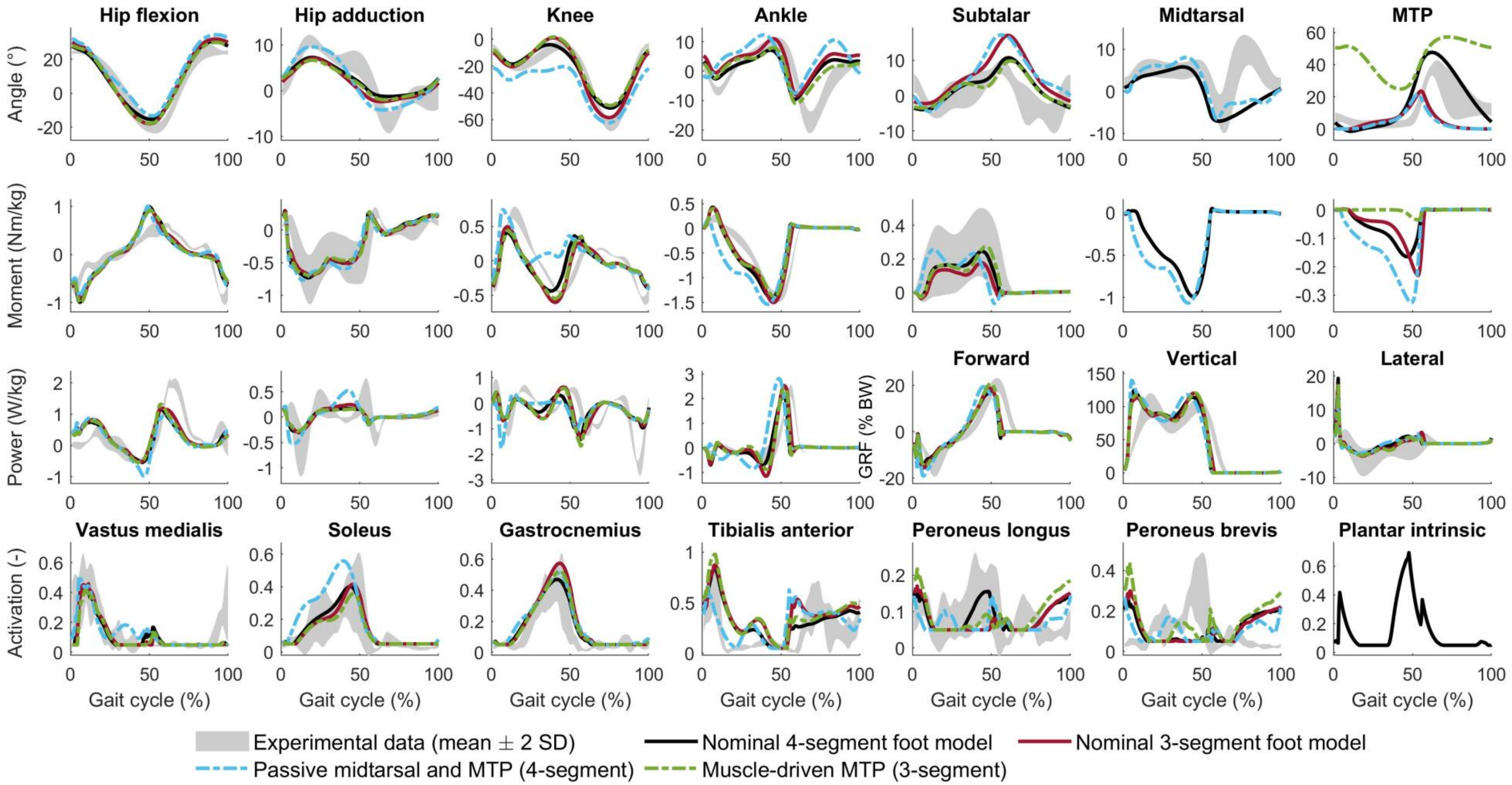

Fig W Effect of extrinsic foot muscles.

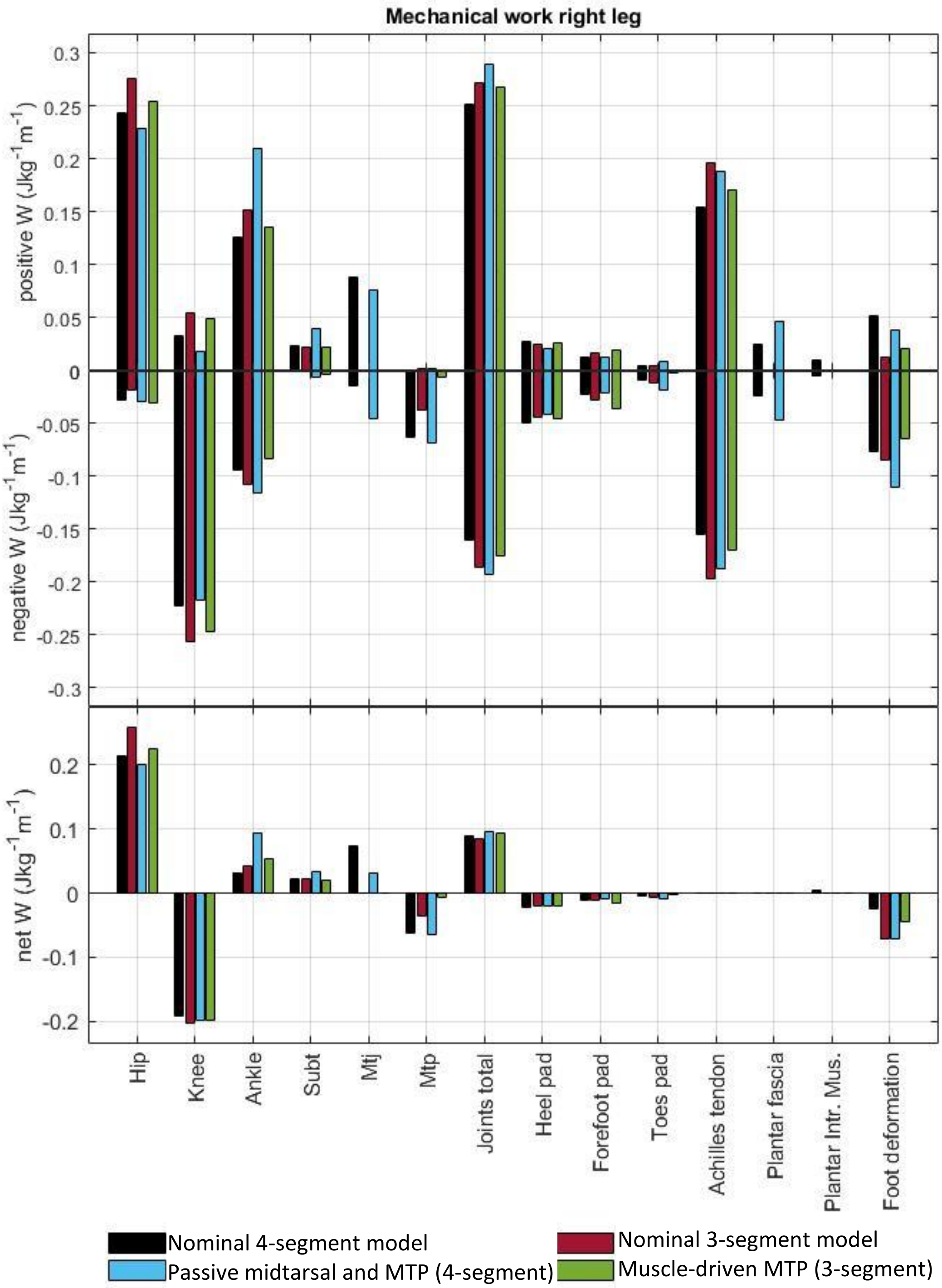

Fig X Effect of extrinsic foot muscles on mechanical work.

8. Approximating conditional statements with a hyperbolic tangent

Elaborate explanation is provided in the supplementary material of (21).

The metabolic energy model (22) contains terms that are not continuously differentiable, such as the work rate of a muscle fibre

$$\dot{W} = \begin{cases} -F^T \cdot v^M \text{ , } if\ v^M \leq 0 \\ 0 \text{ , } if\ v^M > 0 \end{cases}$$

To make this model compatible with algorithmic differentiation, it was approximated as

$$\dot{W} = -F^T \cdot v^M \cdot (0{,}5 - 0{,}5 \tanh(b \cdot v^M))$$

$$\dot{W} = -F^T \cdot v^M_{negative}$$

The smoothing coefficient (b) determines how smooth the transition between the conditions is. A lower value results in a smoother curve, however also leads to distortion in a larger range of values around 0 (Fig Y). Since using $b = 10$ (cfr. (3)) leads to distortion of the negative fibre velocities, and thus an underrepresentation of work rate in metabolic energy rate, we opted to use $b = 100$. Overall effects on simulated gait are limited, thus previous results remain valid.

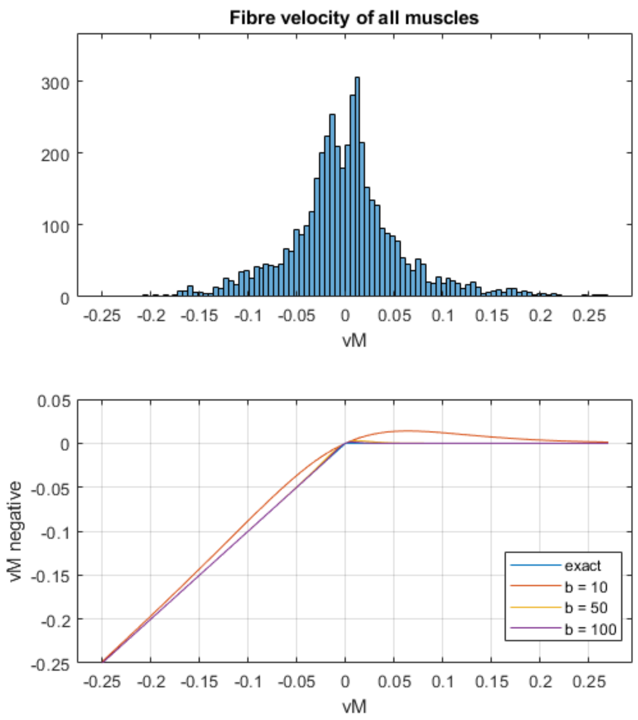

Fig Y Top: histogram of muscle fibre velocities for all muscles over a simulated stride (100 samples over stride duration). Bottom: Smoothed versus exact negative fibre velocity. A low smoothing coefficient (b = 10) distorts the negative contraction velocities that are used to calculate work rate of the muscle fibre.

9. Convergence analysis

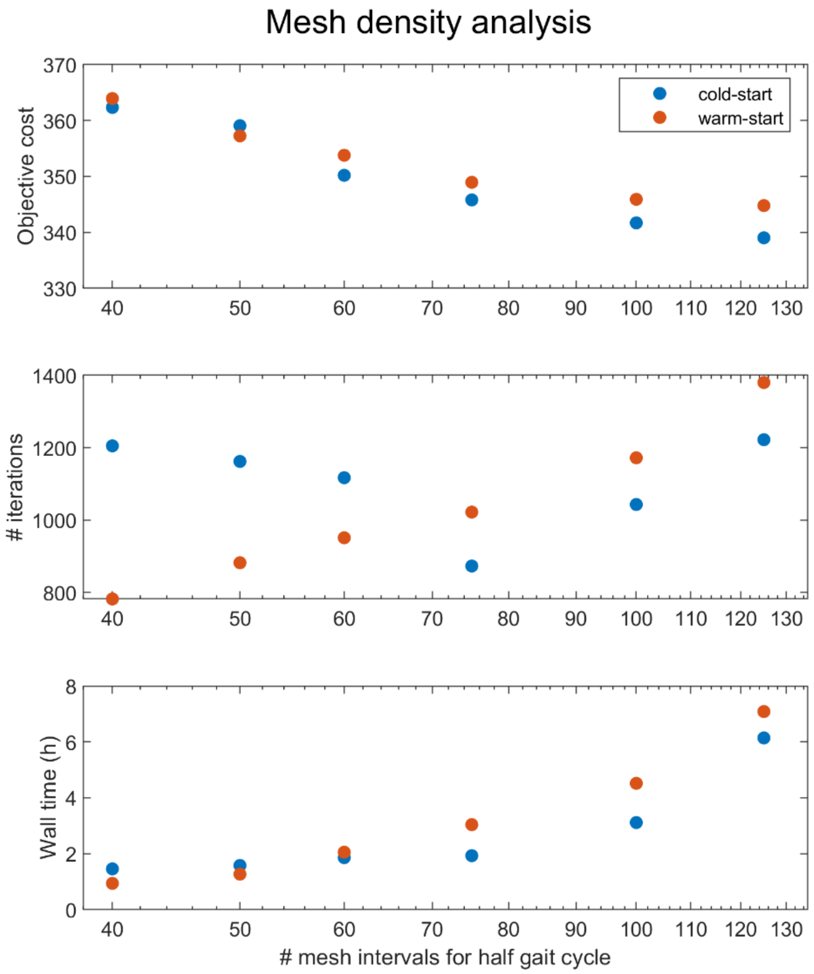

Fig Z We simulated gait for the nominal 4-segment foot model with different amounts of time mesh intervals and initial guesses. Cold-start (i.e. without any reference gait data) resulted in lower objective cost overall. Refining the mesh further than 100 intervals did not yield a relevant improvement in objective cost (1% improvement), thus we selected 100 mesh intervals with cold-start initial guess for all simulations shown.

Table B Reducing convergence tolerance of the optimisation solver below 1e-04 has a negligible effect on optimal cost.

IPOPT tolerance	Optimal cost
1e-04	341.693
1e-05	341.506
1e-06	341.506

10. Marker protocol

Table C Marker placement for the feet.
The marker protocol is a subset of the markers used by Boey et al. (23).

Segment	Marker	Anatomical position
Hindfoot	Hindfoot 1<br>Hindfoot 2<br>Hindfoot 3<br>Hindfoot 4	Craniodorsal calcaneus<br>Caudal dorsal calcaneus<br>Ventrolateral calcaneus<br>Ventromedial calcaneus
Forefoot	Forefoot 1<br>Forefoot 3<br>Forefoot 4<br>Forefoot 6	Basis of fifth metatarsal<br>Basis of first metatarsal<br>Head of fifth metatarsal<br>Head of first metatarsal
Toes	Toes 1<br>Toes 2	Hallux<br>Third toe

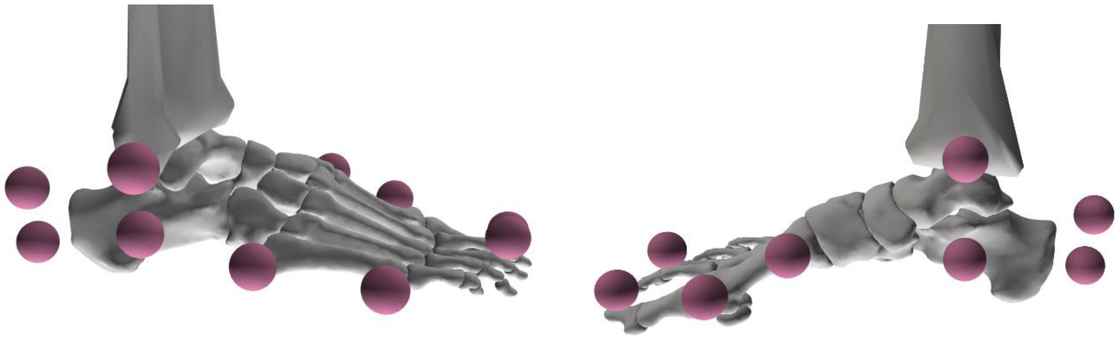

Fig AA Visualisation of all foot markers (Table C) and markers on the malleoli. Visualised via OpenSim (12,13).

11. Predictive gait simulations with 3-segment and 4-segment foot models

Cross-correlation coefficients

Good agreement in bold, moderate agreement in italic.

Table D Cross-correlation coefficients between simulated and mean experimental joint angles (stance phase)

**stance kinematics**	pelvis forward	pelvis vertical	pelvis lateral	pelvis tilt	pelvis list	pelvis rotation	hip flexion	hip adduction	hip rotation	knee	ankle	subtalar	midtarsal	MTP	lumbar extension	lumbar bending	lumbar rotation
3-segment foot model Falisse et al. (21)	**1.00**	**0.98**	*0.81*	-0.18	0.64	0.19	**0.99**	**0.94**	0.19	**0.94**	0.32	-0.04		*0.89*	**0.99**	*0.86*	0.39
new 3-segment foot model	**1.00**	**0.98**	0.76	-0.16	0.77	0.14	**0.99**	**0.97**	0.04	**0.95**	0.76	*0.87*		**0.94**	**0.99**	**0.90**	0.59
4-segment foot model	**1.00**	**0.98**	0.67	-0.15	0.74	0.11	**0.99**	**0.97**	0.01	**0.99**	**0.91**	*0.82*	**0.97**	**0.97**	**0.98**	**0.92**	0.49

Table E Cross-correlation coefficients between simulated and mean experimental joint angles (swing phase)

**swing kinematics**	pelvis forward	pelvis vertical	pelvis lateral	pelvis tilt	pelvis list	pelvis rotation	hip flexion	hip adduction	hip rotation	knee	ankle	subtalar	midtarsal	MTP	lumbar extension	lumbar bending	lumbar rotation
3-segment foot model Falisse et al. (21)	**1.00**	**1.00**	*0.71*	-0.36	*0.81*	*0.77*	**0.98**	*0.89*	0.32	**0.99**	0.04	-0.07		0.82	**0.99**	**0.94**	0.27
new 3-segment foot model	**1.00**	**1.00**	0.61	-0.34	*0.86*	*0.79*	**0.98**	*0.87*	0.47	**0.98**	0.58	-0.42		0.80	**1.00**	**0.91**	0.51
4-segment foot model	**1.00**	**1.00**	0.44	-0.33	*0.82*	*0.81*	**0.96**	0.56	0.45	**0.97**	*0.80*	-0.32	-0.75	**0.97**	**0.99**	**0.93**	0.43

Table F Cross-correlation coefficients between simulated and mean experimental joint moments (stance phase)

**stance kinetics**	hip flexion	hip adduction	hip rotation	knee	ankle	subtalar	lumbar extension	lumbar bending	lumbar rotation
3-segment foot model Falisse et al. (21)	**0.92**	**0.93**	**0.92**	**0.90**	**0.97**	**0.92**	-0.11	*0.84*	-0.30
new 3-segment foot model	**0.94**	**0.94**	**0.95**	*0.88*	**0.96**	**0.96**	-0.17	*0.85*	0.09
4-segment foot model	**0.93**	**0.92**	**0.92**	*0.86*	**0.97**	**0.97**	-0.06	*0.82*	0.06

Table G Cross-correlation coefficients between simulated and mean experimental joint moments (swing phase)

**swing kinetics**	hip flexion	hip adduction	hip rotation	knee	ankle	subtalar	lumbar extension	lumbar bending	lumbar rotation
3-segment foot model Falisse et al. (21)	*0.89*	*0.80*	0.20	**0.92**	0.58	-0.19	0.45	*0.82*	-0.43
new 3-segment foot model	*0.82*	*0.83*	*0.89*	*0.89*	*0.81*	-0.30	0.24	*0.83*	0.42
4-segment foot model	*0.84*	*0.80*	*0.86*	*0.86*	*0.82*	-0.19	0.37	*0.84*	0.22

Table H Cross-correlation coefficients between simulated and mean experimental joint powers (stance phase)

**stance energetics**	hip flexion	hip adduction	hip rotation	knee	ankle	subtalar	lumbar extension	lumbar bending	lumbar rotation
3-segment foot model Falisse et al. (21)	*0.70*	0.55	0.08	*0.80*	0.65	0.68	-0.28	0.21	-0.27
new 3-segment foot model	0.69	0.63	-0.06	*0.84*	*0.74*	*0.71*	-0.39	0.59	0.11
4-segment foot model	0.50	0.63	-0.04	*0.85*	*0.83*	0.38	-0.25	0.57	-0.08

Table I Cross-correlation coefficients between simulated and mean experimental joint powers (swing phase)

**swing energetics**	hip flexion	hip adduction	hip rotation	knee	ankle	subtalar	lumbar extension	lumbar bending	lumbar rotation
3-segment foot model Falisse et al. (21)	**0.96**	0.54	-0.68	*0.85*	0.38	0.38	-0.26	0.23	-0.31
new 3-segment foot model	**0.97**	0.56	-0.17	*0.82*	*0.78*	0.06	-0.16	0.55	0.21
4-segment foot model	**0.97**	0.54	-0.13	*0.82*	*0.76*	-0.45	-0.14	0.66	-0.01

Weighted root mean square errors

Good agreement in bold, moderate agreement in italic.

Table J RMSE between simulated and mean experimental joint angles (stance phase)

**stance kinematics**	pelvis forward	pelvis vertical	pelvis lateral	pelvis tilt	pelvis list	pelvis rotation	hip flexion	hip adduction	hip rotation	knee	ankle	subtalar	midtarsal	MTP	lumbar extension	lumbar bending	lumbar rotation
3-segment foot model Falisse et al. (21)	**1.27**	**1.43**	**0.93**	3.66	4.87	**1.84**	4.50	*2.18*	5.40	4.60	11.98	7.24		5.23	**1.94**	**1.49**	**1.83**
new 3-segment foot model	**1.27**	*2.95*	**0.53**	3.70	4.25	**1.57**	3.88	3.23	5.17	3.72	5.38	*2.93*		3.22	3.94	**1.60**	**1.76**
4-segment foot model	*2.49*	3.56	**0.56**	3.74	4.61	**1.36**	*2.52*	*2.84*	5.26	*2.56*	4.15	*2.12*	**1.20**	*2.29*	3.52	**1.72**	**1.82**

Table K RMSE between simulated and mean experimental joint angles (swing phase)

**swing kinematics**	pelvis forward	pelvis vertical	pelvis lateral	pelvis tilt	pelvis list	pelvis rotation	hip flexion	hip adduction	hip rotation	knee	ankle	subtalar	midtarsal	MTP	lumbar extension	lumbar bending	lumbar rotation
3-segment foot model Falisse et al. (21)	*2.08*	**0.88**	**0.37**	*2.39*	3.79	**1.57**	6.55	**1.53**	*2.65*	4.48	11.51	*2.98*		3.98	**1.15**	3.14	*2.45*
new 3-segment foot model	*2.41*	*2.05*	**0.33**	*2.43*	3.60	**1.27**	5.67	*2.15*	*3.00*	4.72	5.54	5.92		4.22	*2.30*	3.45	*2.29*
4-segment foot model	**1.69**	*2.05*	**0.38**	*2.46*	3.86	**1.03**	4.58	*2.95*	3.29	5.34	3.76	4.10	5.14	*2.67*	*2.06*	3.72	*2.36*

Table L RMSE between simulated and mean experimental joint moments (stance phase)

**stance kinetics**	hip flexion	hip adduction	hip rotation	knee	ankle	subtalar	lumbar extension	lumbar bending	lumbar rotation
3-segment foot model Falisse et al. (21)	5.15	2.76	3.67	5.13	18.56	16.44	7.39	4.69	6.52
new 3-segment foot model	5.27	3.20	3.08	7.01	18.36	**1.88**	6.39	4.34	6.34
4-segment foot model	5.95	3.71	3.79	7.52	11.28	**1.29**	6.37	4.64	6.53

Table M RMSE between simulated and mean experimental joint moments (swing phase)

**swing kinetics**	hip flexion	hip adduction	hip rotation	knee	ankle	subtalar	lumbar extension	lumbar bending	lumbar rotation
3-segment foot model Falisse et al. (21)	10.73	3.35	3.96	12.13	27.41	13.42	3.77	4.63	6.50
new 3-segment foot model	11.96	3.83	4.31	13.59	14.98	3.76	4.98	5.74	6.02
4-segment foot model	11.75	4.15	4.17	16.78	13.93	3.55	4.55	4.76	6.08

Table N RMSE between simulated and mean experimental joint powers (stance phase)

**stance energetics**	hip flexion	hip adduction	hip rotation	knee	ankle	subtalar	lumbar extension	lumbar bending	lumbar rotation
3-segment foot model Falisse et al. (21)	11.20	7.50	8.20	5.43	14.86	5.34	3.19	5.66	3.86
new 3-segment foot model	9.71	5.36	6.48	6.43	14.27	**1.79**	*2.92*	4.84	3.81
4-segment foot model	10.05	4.59	5.96	6.01	8.53	*2.61*	*2.78*	5.01	3.84

Table O RMSE between simulated and mean experimental joint powers (swing phase)

**swing energetics**	hip flexion	hip adduction	hip rotation	knee	ankle	subtalar	lumbar extension	lumbar bending	lumbar rotation
3-segment foot model Falisse et al. (21)	4.16	3.71	2.15	8.28	8.36	3.31	**1.70**	15.71	3.75
new 3-segment foot model	3.98	2.54	**1.62**	8.42	5.98	3.12	**1.88**	10.80	3.71
4-segment foot model	4.45	3.19	**1.47**	8.84	6.98	3.22	*2.14*	9.09	3.73

Additional Figs

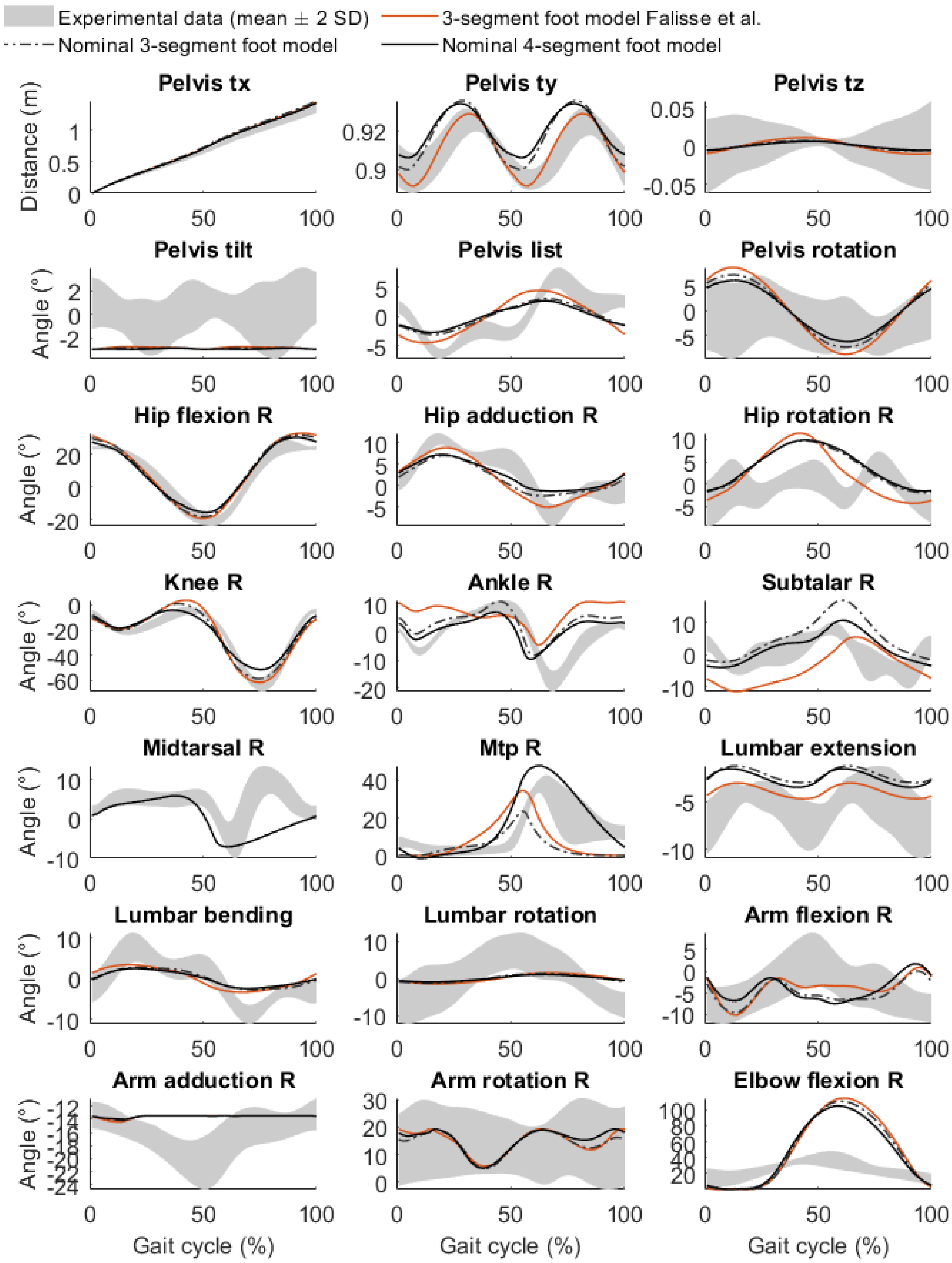

Fig AB All kinematics (right side) for gait simulations with our nominal 4-segment foot model, 3-segment foot model, and the model from Falisse et al. (21)

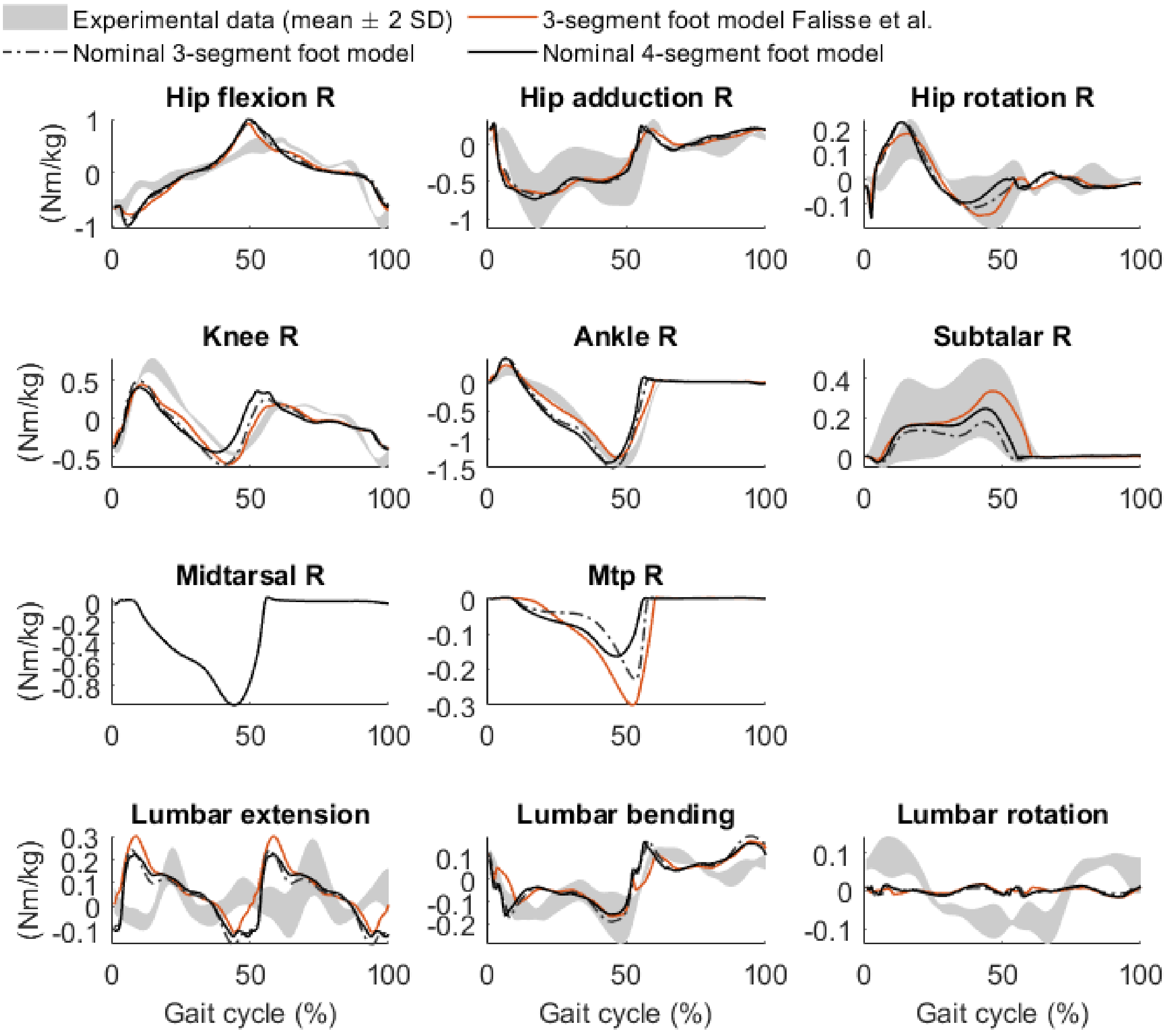

Fig AC Joint moments of muscle-driven joints (right side) for gait simulations with our nominal 4-segment foot model, 3-segment foot model, and the model from Falisse et al. (21)

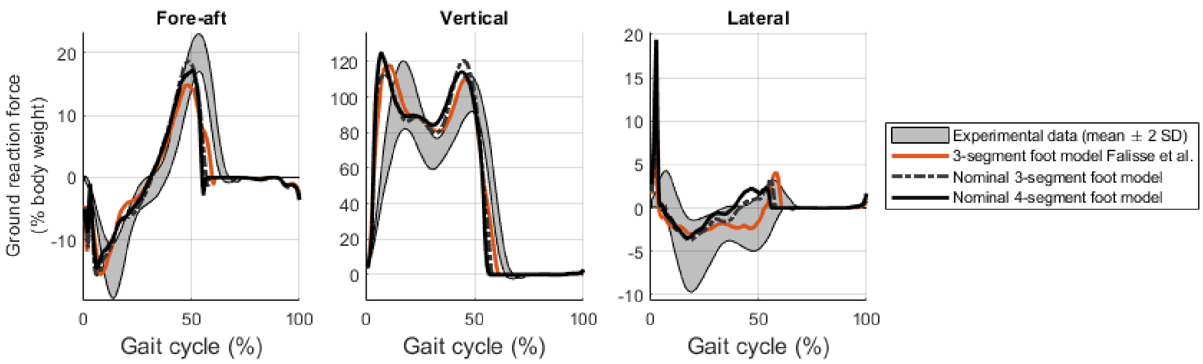

Fig AD Ground reaction forces

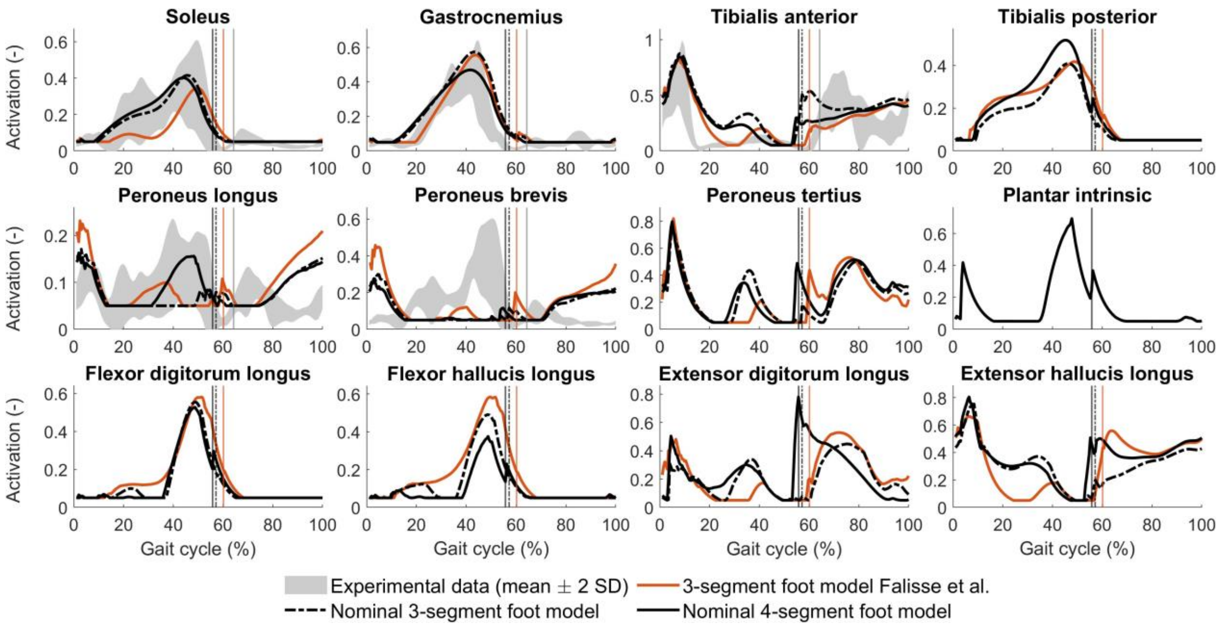

Fig AE Activation of ankle-foot muscles. Gastrocnemius indicates the medial gastrocnemius. Medial and lateral gastrocnemius activation patterns were equivalent.

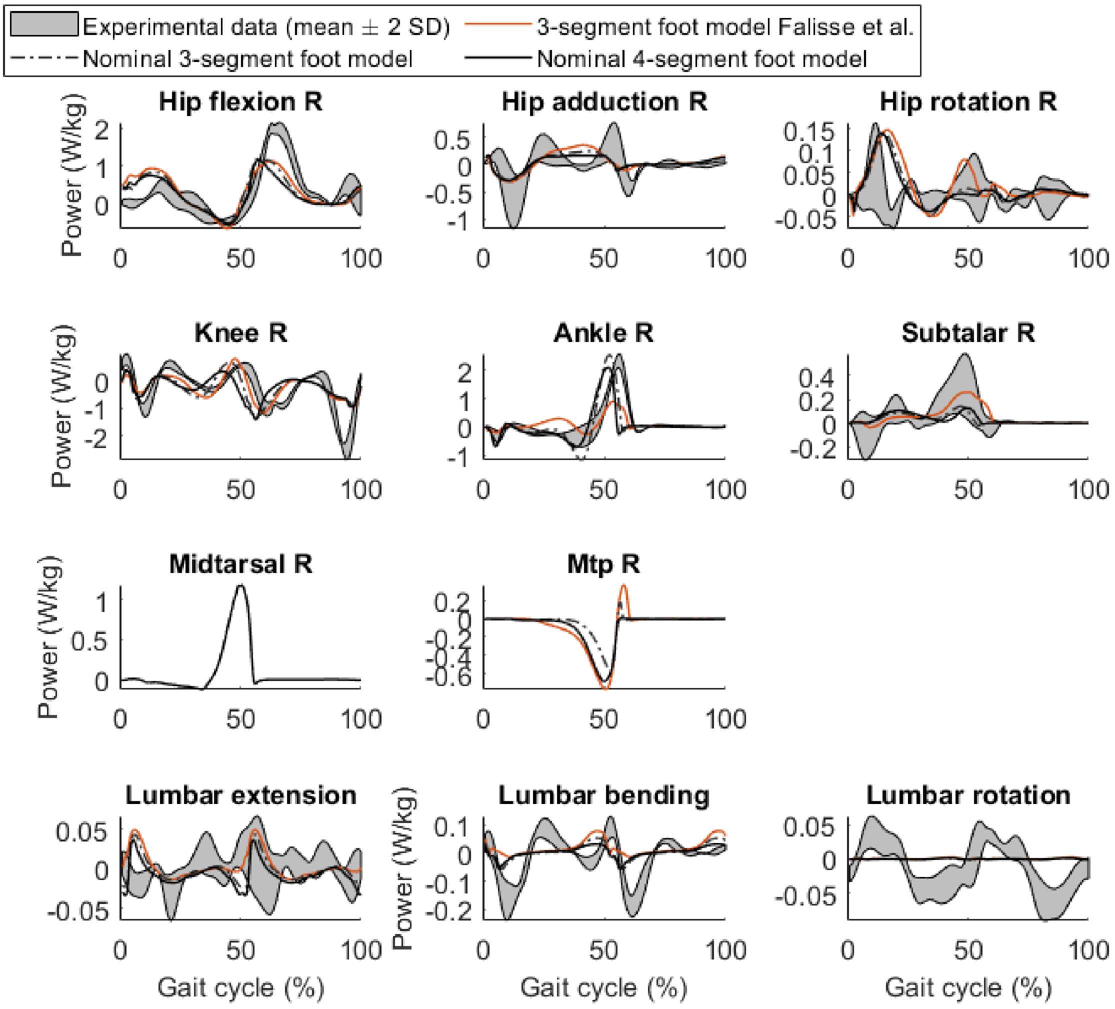

Fig AF Joint powers of muscle-driven joints (right side) for gait simulations with our nominal 4-segment foot model, 3-segment foot model, and the model from Falisse et al. (21)

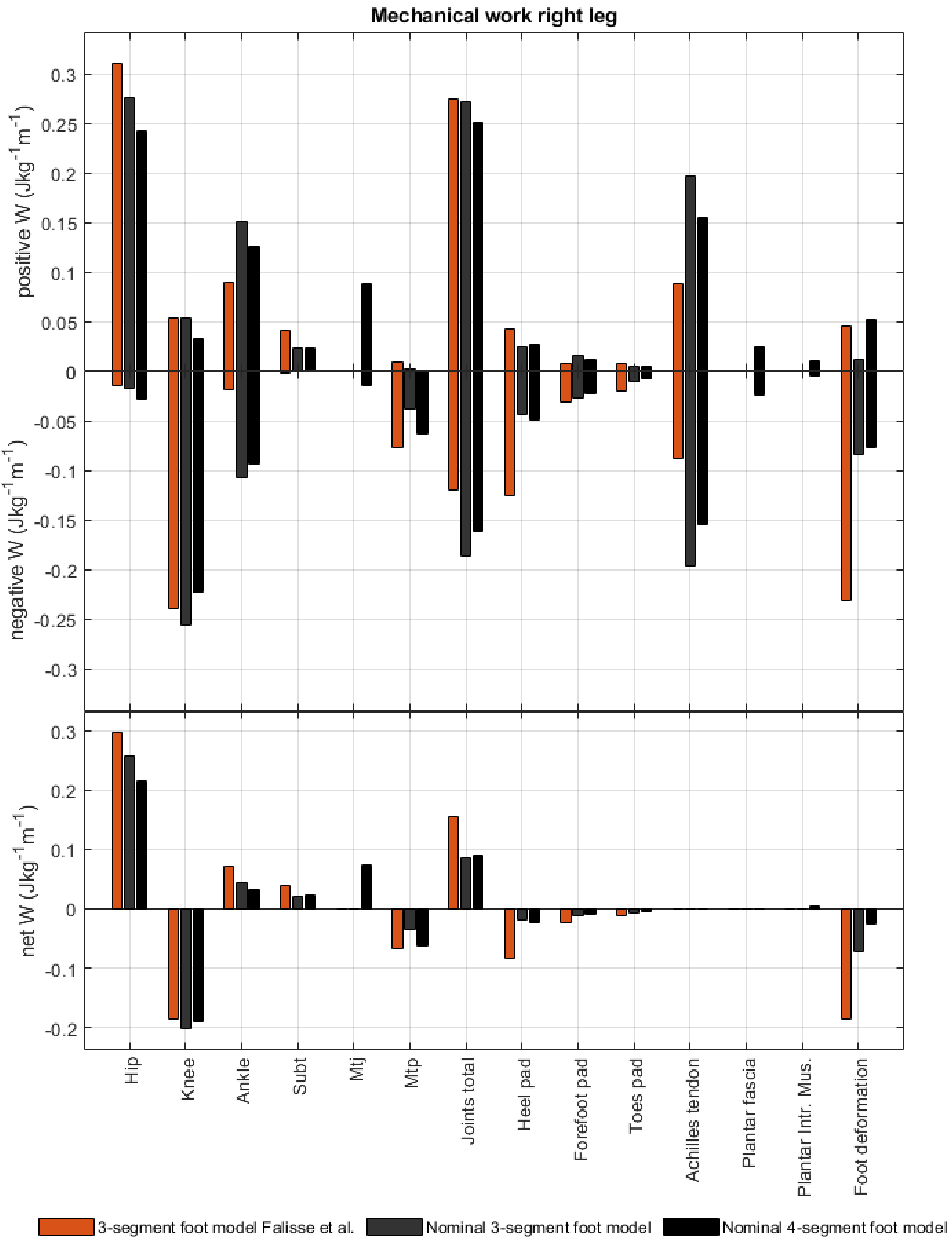

Fig AG Positive, negative, and net mechanical work around joints and by selected structures. Positive net work indicates energy generation, negative energy dissipation.

12. Reducing the ability to stiffen the foot

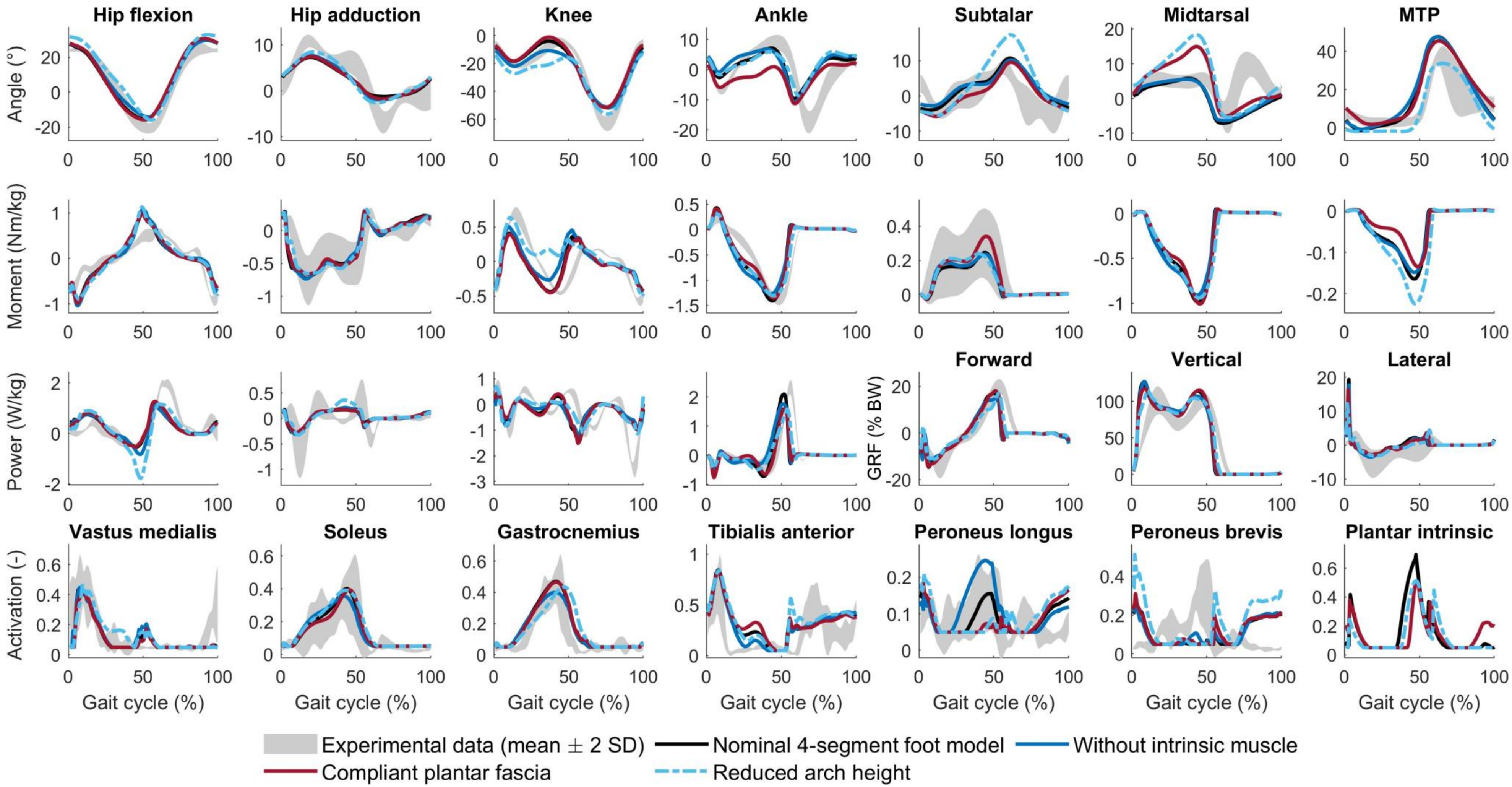

Fig AH We simulated a model without intrinsic foot muscles, with a compliant plantar fascia and with a reduced arch height to evaluate the effect of the ability to stiffen the foot arch on the predicted walking motion.

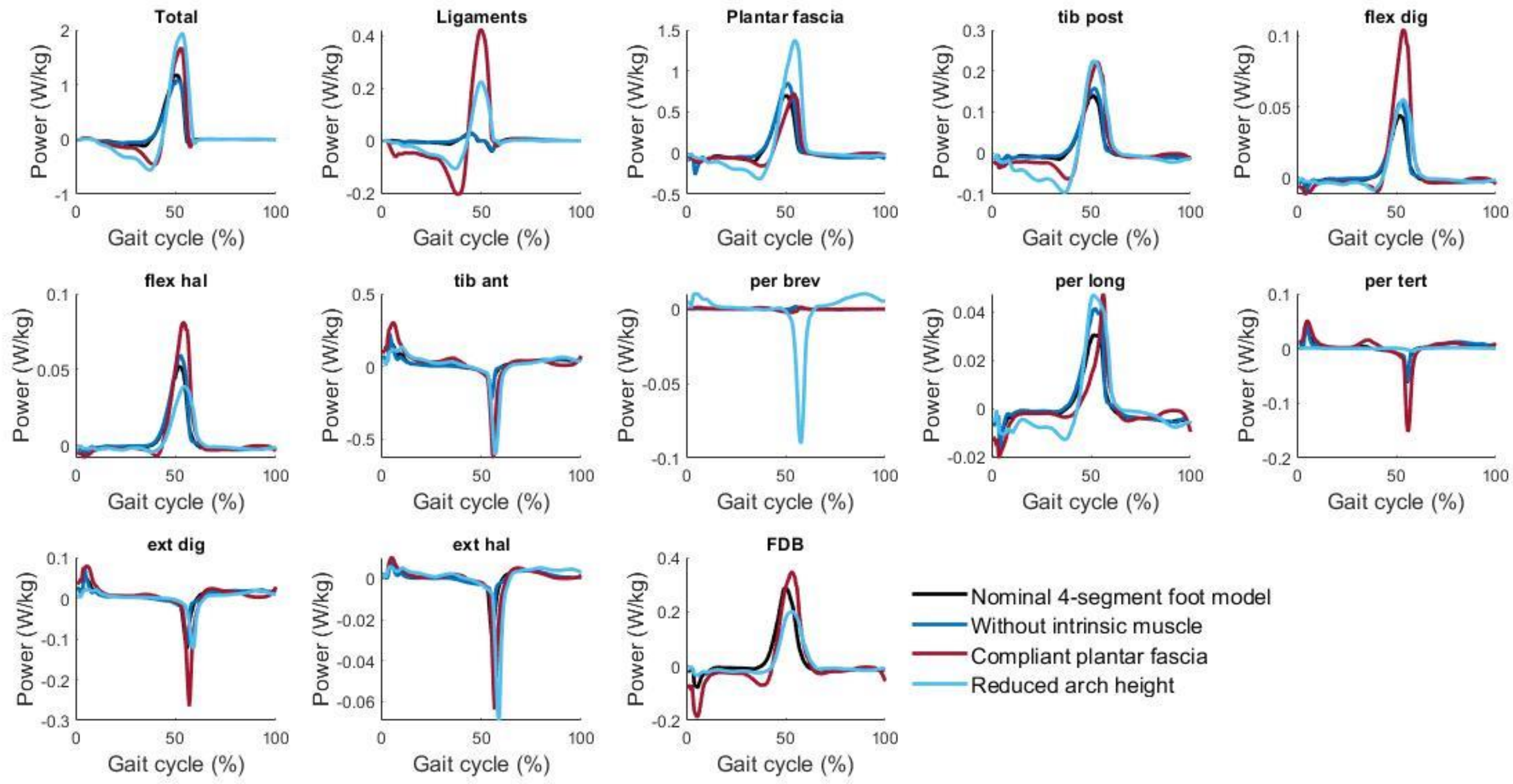

Fig AI Contribution of different structures to midtarsal joint power. Plantar intrinsic muscle is indicated by FDB (Flexor Digitorum Brevis).

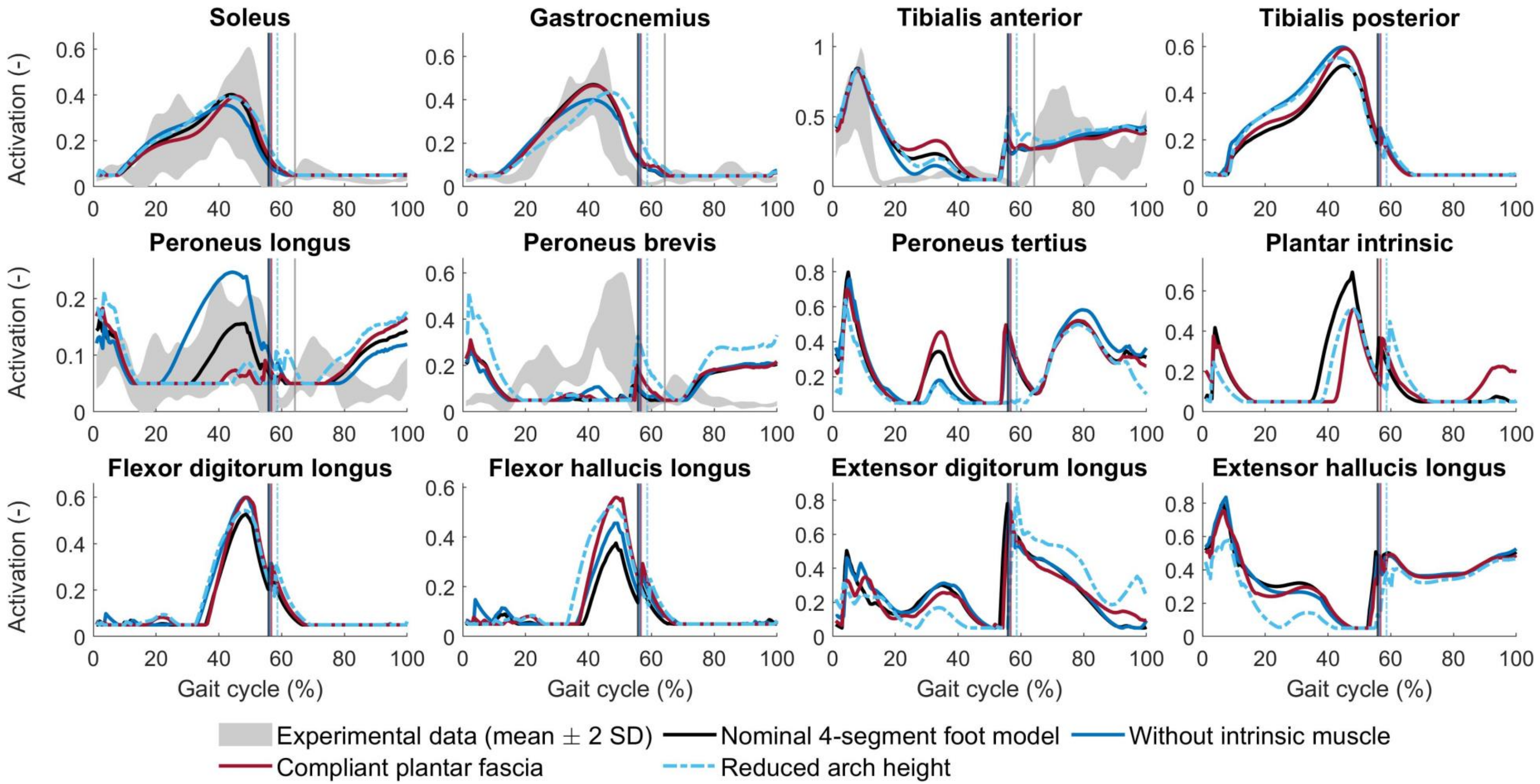

Fig AJ Activation of ankle-foot muscles. Gastrocnemius indicates the medial gastrocnemius, lateral gastrocnemius activation patterns are equivalent.

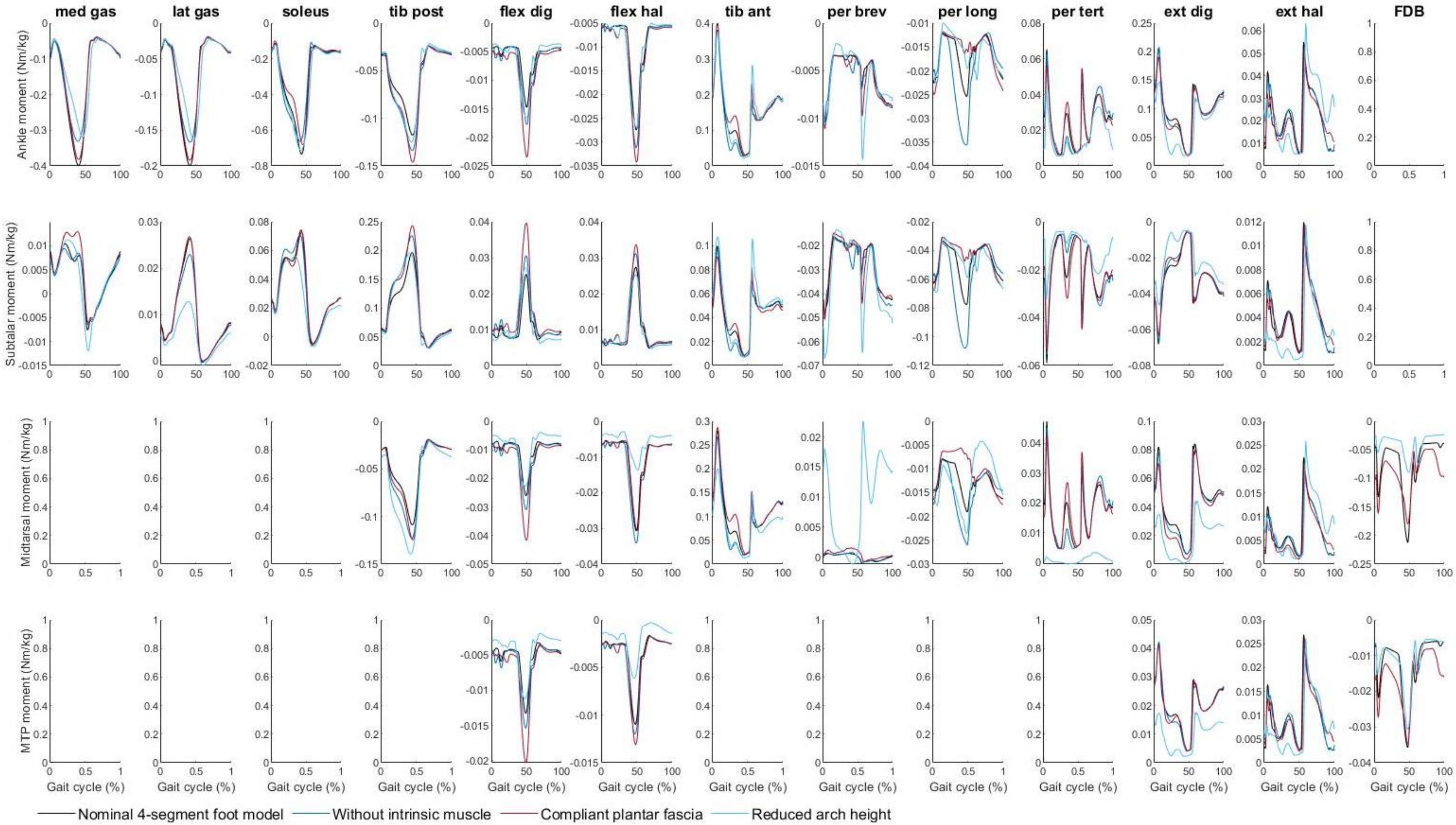

Fig AK Ankle-foot joint moments generated by each muscle. Each row represents a joint, each column a muscle. Empty plots indicate that the muscle does not span the joint. Plantar intrinsic muscle is indicated by FDB (Flexor Digitorum Brevis).

13. Foot arch height

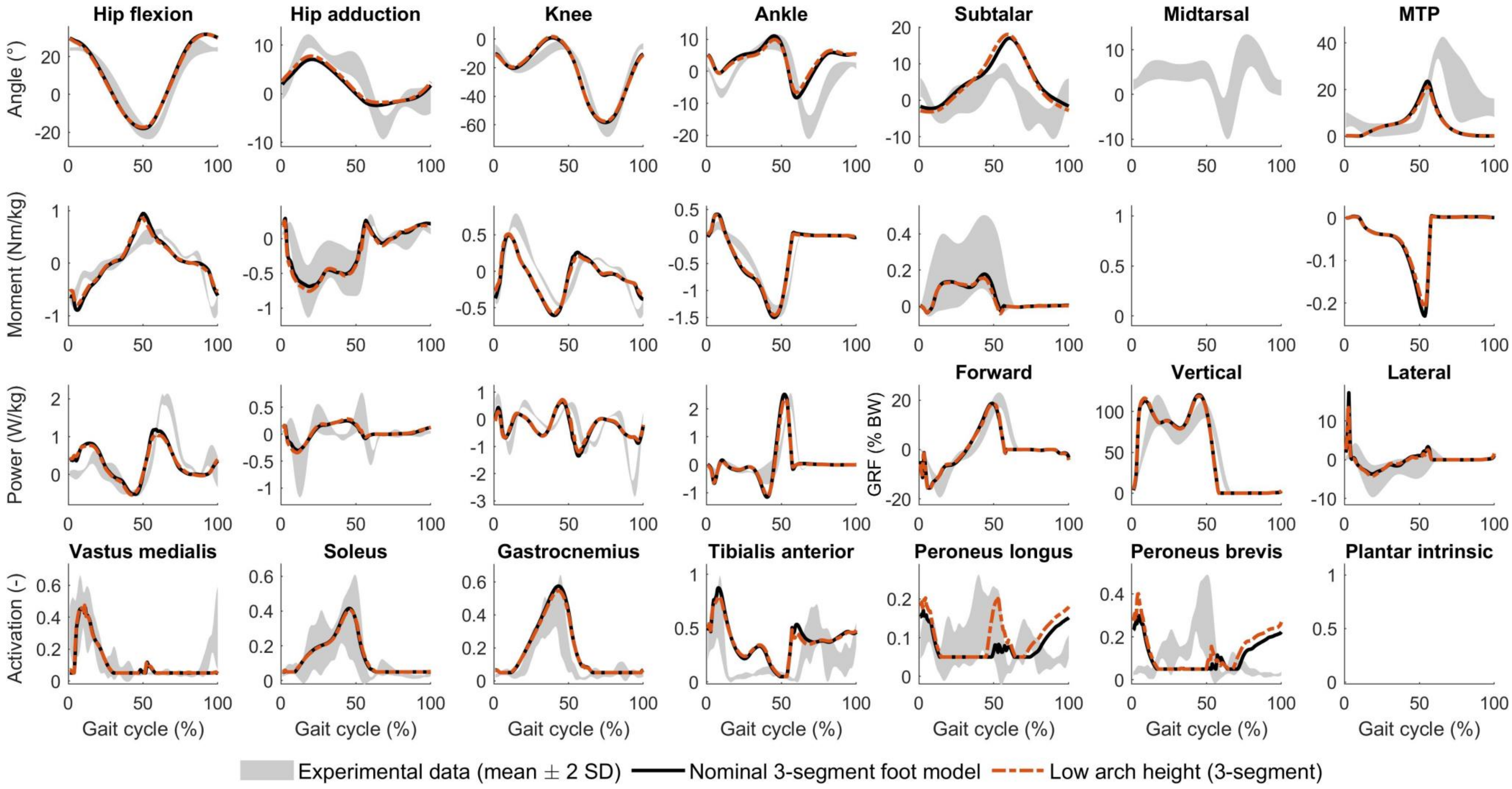

Fig AL Effect of foot arch height on gait, for the 3-segment foot model.

14. Plantar intrinsic muscle nerve block

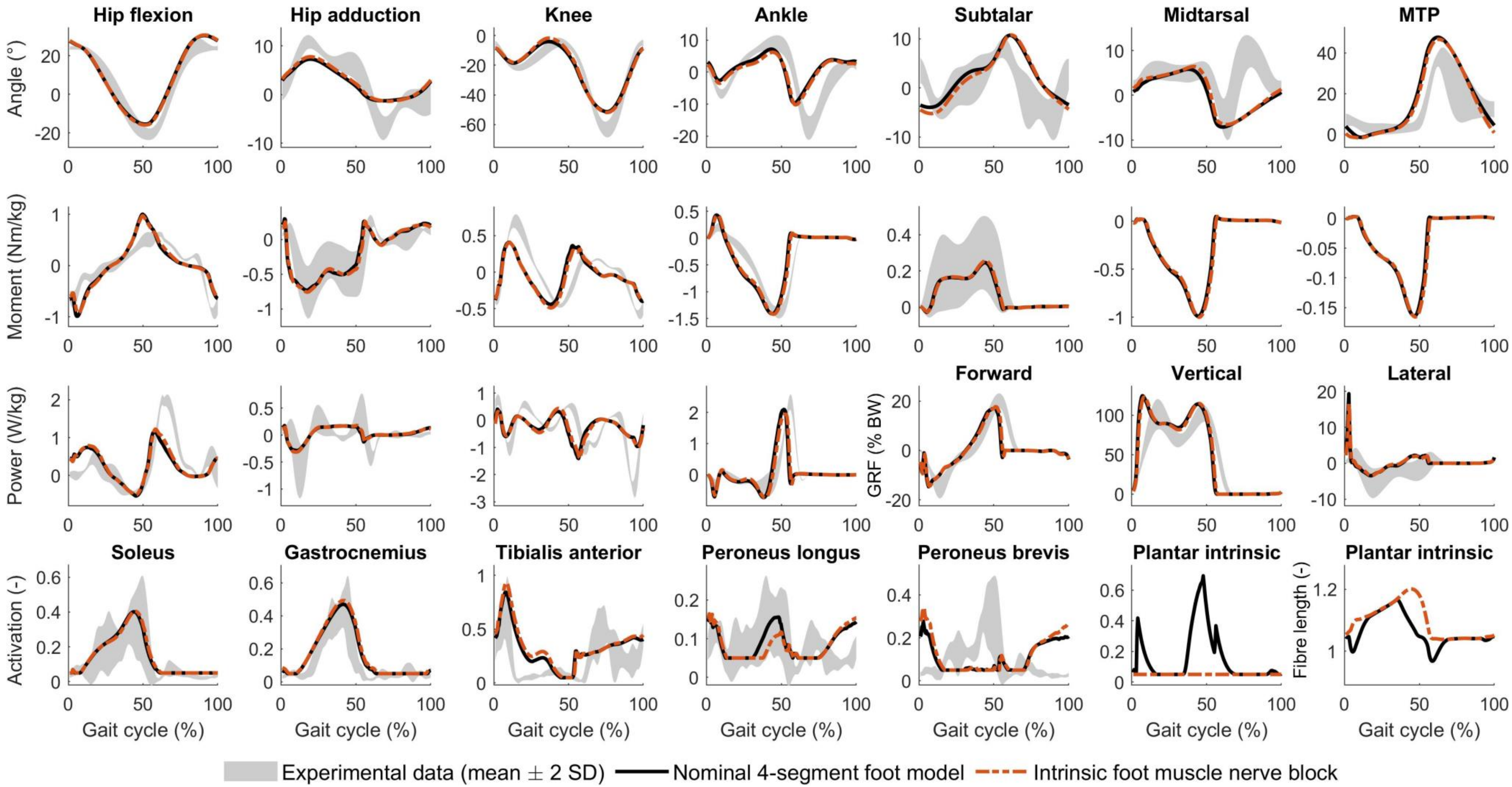

Fig AM Effect of intrinsic muscle nerve block on gait.

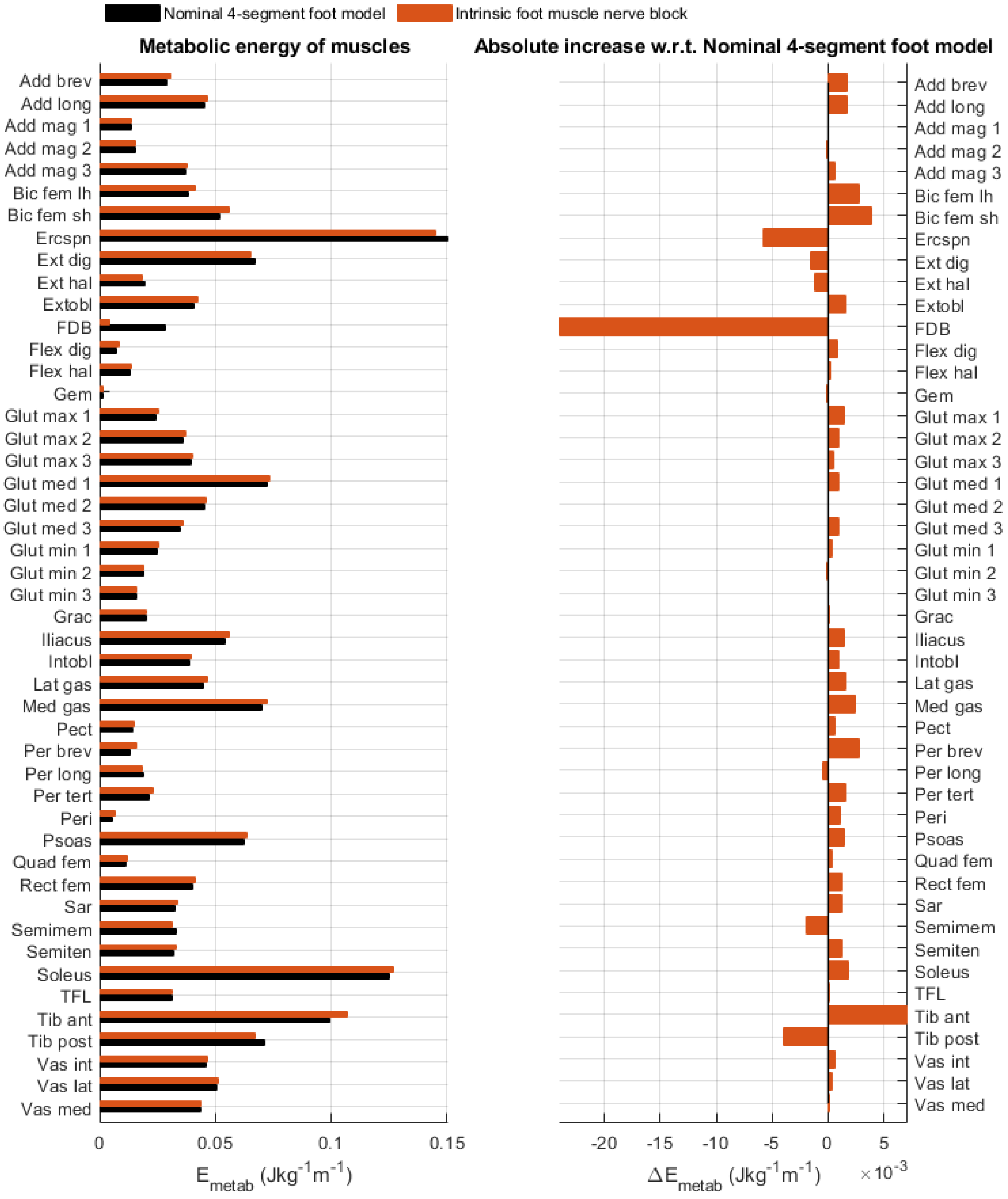

Fig AN Metabolic energy expenditure of every muscle (right side). Energy is normalised to body mass and distance travelled (cfr. Cost of transport). Left graph shows total values, right graph shows difference with nominal 4-segment foot model. The plantar intrinsic foot muscle is indicated by FDB (flexor digitorum brevis).

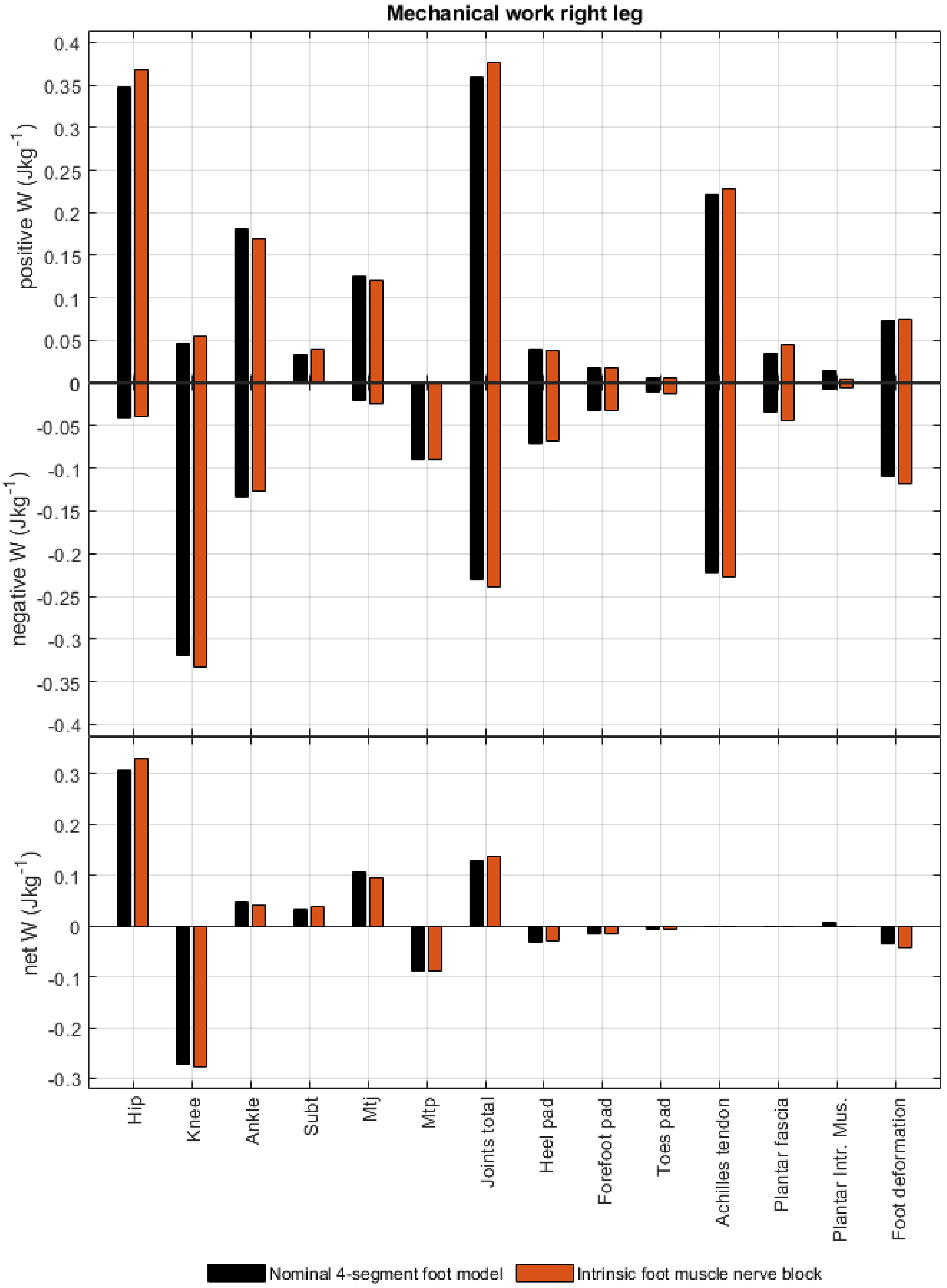

Fig AO Effect of intrinsic muscle nerve block on positive, negative, and net work around joints and by selected structures.

References

1. De Groote F, Kinney AL, Rao AV, Fregly BJ. Evaluation of Direct Collocation Optimal Control Problem Formulations for Solving the Muscle Redundancy Problem. Ann Biomed Eng. 2016;44(10):2922–36.

2. Nordez A, Gross R, Andrade R, Le Sant G, Freitas S, Ellis R, et al. Non-Muscular Structures Can Limit the Maximal Joint Range of Motion during Stretching. Sports Med. 2017 Oct 1;47(10):1925–9.

3. Falisse A, Serrancoli G, Dembia CL, Gillis J, Jonkers I, De Groote F. Rapid predictive simulations with complex musculoskeletal models suggest that diverse healthy and pathological human gaits can emerge from similar control strategies. J R Soc Interface. 2019;16(157):20190402.

4. Anderson DE, Madigan ML, Nussbaum MA. Maximum voluntary joint torque as a function of joint angle and angular velocity: Model development and application to the lower limb. J Biomech. 2007 Jan 1;40(14):3105–13.

5. Marsh E, Sale D, McComas AJ, Quinlan J. Influence of joint position on ankle dorsiflexion in humans. J Appl Physiol. 1981 Jul;51(1):160–7.

6. Sale D, Quinlan J, Marsh E, McComas AJ, Belanger AY. Influence of joint position on ankle plantarflexion in humans. J Appl Physiol. 1982 Jun;52(6):1636–42.

7. Holzer D, Paternoster FK, Hahn D, Siebert T, Seiberl W. Considerations on the human Achilles tendon moment arm for in vivo triceps surae muscle–tendon unit force estimates. Sci Rep. 2020 Nov 11;10(1):19559.

8. Malaquias TM, Silveira C, Aerts W, De Groote F, Dereymaeker G, Vander Sloten J, et al. Extended foot-ankle musculoskeletal models for application in movement analysis. Comput Methods Biomech Biomed Engin. 2017;20(2):153–9.

9. Caravaggi P, Pataky T, Goulermas JY, Savage R, Crompton R. A dynamic model of the windlass mechanism of the foot: Evidence for early stance phase preloading of the plantar aponeurosis. J Exp Biol. 2009;212(15):2491–9.

10. De Groote I, Humphrey LT. Body mass and stature estimation based on the first metatarsal in humans. Am J Phys Anthropol. 2011;144(4):625–32.

11. Ancillao A. The helical axis of anatomical joints: calculation methods, literature review, and software implementation. Med Biol Eng Comput. 2022 Jul 1;60(7):1815–25.

12. Delp SL, Anderson FC, Arnold AS, Loan P, Habib A, John CT, et al. OpenSim: Open-Source Software to Create and Analyze Dynamic Simulations of Movement. IEEE Trans Biomed Eng. 2007;54(11):1940–50.

13. Seth A, Hicks JL, Uchida TK, Habib A, Dembia CL, Dunne JJ, et al. OpenSim: Simulating musculoskeletal dynamics and neuromuscular control to study human and animal movement. PLOS Comput Biol. 2018 Jul 26;14(7):e1006223.

14. Gefen A. Stress analysis of the standing foot following surgical plantar fascia release. J Biomech. 2002;35(5):629–37.

15. Kitaoka HB, Luo ZP, Growney ES, Berglund LJ, An KN. Material Properties of the Plantar Aponeurosis. Foot Ankle Int. 1994 Oct 1;15(10):557–60.

16. Fessel G, Jacob HAC, Wyss Ch, Mittlmeier Th, Müller-Gerbl M, Büttner A. Changes in length of the plantar aponeurosis during the stance phase of gait – An in vivo dynamic fluoroscopic study. Ann Anat - Anat Anz. 2014 Dec 1;196(6):471–8.

17. Wright DG, Rennels DC. A Study of the Elastic Properties of Plantar Fascia: J Bone Jt Surg. 1964 Apr;46(3):482–92.

18. Chen DW, Li B, Aubeeluck A, Yang YF, Huang YG, Zhou JQ, et al. Anatomy and biomechanical properties of the plantar aponeurosis: A cadaveric study. PloS One. 2014;9(1):e84347–e84347.

19. Natali AN, Pavan PG, Stecco C. A constitutive model for the mechanical characterization of the plantar fascia. Connect Tissue Res. 2010;51(5):337–46.

20. Serrancolí G, Falisse A, Dembia C, Vantilt J, Tanghe K, Lefeber D, et al. Subject-Exoskeleton Contact Model Calibration Leads to Accurate Interaction Force Predictions. IEEE Trans Neural Syst Rehabil Eng. 2019 Aug;27(8):1597–605.

21. Falisse A, Afschrift M, Groote FD. Modeling toes contributes to realistic stance knee mechanics in three-dimensional predictive simulations of walking. PLOS ONE. 2022 Jan 25;17(1):e0256311.

22. Bhargava LJ, Pandy MG, Anderson FC. A phenomenological model for estimating metabolic energy consumption in muscle contraction. J Biomech. 2004;37(1):81–8.

23. Boey H, van Rossom S, Verfaillie S, Sloten JV, Jonkers I. Maximal lateral ligament strain and loading during functional activities: Model-based insights for ankle sprain prevention and rehabilitation. Clin Biomech. 2022 Apr 1;94:105623.
