# Supplementary material for: A dynamic foot model for predictive simulations of human gait reveals causal relations between foot structure and whole-body mechanics
Source: PLoS Comput Biol. 2024 Jun 20;20(6):e1012219. doi: 10.1371/journal.pcbi.1012219 (PMC11218950; doi:10.1371/journal.pcbi.1012219)
Supplement: Attachment — Submitted filename: DHondt2024_response_to_reviewers.docx [file pcbi.1012219.s002.docx]

**Response to review**

**General comments to reviewer 1 and 2**

We thank the reviewers for their thoughtful reviews that helped us to strengthen the manuscript.

We have updated the naming convention of our models to include the talus when counting the number of foot segments. We had used two-segment and three-segment to be consistent with previous work from our lab (Falisse et al, 2022). This does not include the talus when counting foot segments, which might be confusing. We changed the names to three-segment and four-segment foot, as this reflects the actual number of rigid-body segments in the foot.

In this document, reviewer questions are in blue. Text from the manuscript is *italic* with additions and changes *underlined*.

**Reviewer 1:**

**Major comments:**

1. Abstract: A few comments to highlight the impact of the paper better:
1a. Currently, it seems like the predictive simulations were the novel part of the work (since they are mentioned first). It could be better to highlight that a new foot was developed first, which was made to work well with predictive simulations.

We made it explicit that we developed the model, then used it for simulations. We changed

*Here we used predictive simulations to explore causal relationships between foot properties and whole-body gait. Our dynamic three-segment foot model with longitudinal arch improved predictions of gait mechanics compared to two-segment foot models without compliant arch.*

To

*Predictive simulations are a powerful tool to explore causal relationships between musculoskeletal properties and whole-body gait. However, musculoskeletal models used in three-dimensional predictive simulations assume a rigid foot arch, limiting their use for studying how foot structure influences three-dimensional gait mechanics. Here, we developed a four-segment foot model with longitudinal arch for use in predictive simulations.*

1b. It is unclear why current foot models are lacking, and in which ways they don’t capture important details. The first few sentences could be shortened to more succinctly describe the shortcomings of experimental studies to make room for this.

We added how current models for 3D predictive simulations assume a rigid foot arch.

*However, musculoskeletal models used in three-dimensional predictive simulations assume a rigid foot arch, limiting their use for studying how foot structure influences three-dimensional gait mechanics.*

1c. L27: Specifically mentioning which “walking mechanics” were captured would strengthen and highlight the strong validation effort.

We replaced “Walking mechanics” with “ankle and knee kinematics, soleus activation, and ankle power”.

*We identified three properties of the ankle-foot complex that are important to capture ankle and knee kinematics, soleus activation, and ankle power of healthy adults*

1d. L30-L33: Is there a succinct way to highlight why replicating these results in particular opens up new opportunities for understanding foot mechanics?

We included why we tried to replicated these experiments.

*The ability to reproduce these different experiments indicates that our foot model captures the main mechanical properties of the foot.*

2. Introduction structure: Like the abstract, the overall structure of the Introduction seems opposite of the contribution of the paper (i.e., it first discusses predictive simulations rather than the foot). The Author Summary’s structure does a better job of highlighting this. I would suggest following that model, and one way to do so would be the following:
2a. Add a new paragraph that discusses the importance of the foot, what experiments have found so far, and how models can help extend our understanding.

We adapted the first paragraph of the introduction to motivate the development of our model with more emphasis on why the foot is important and what experiments have found so far.

*During locomotion, we interact with the environment through our feet . The feet have a weightbearing function but also transfers the forces needed to accelerate and decelerate the body during movements. The foot’s arched shape enables stiffening the foot to withstand high loads, without compromising the flexibility of the foot (1). A compliant foot enables storage and release of elastic energy in the ligaments crossing foot joints, which might contribute to gait efficiency (2). Intrinsic foot muscles can modulate the visco-elastic properties of the foot to absorb a shock, assist in pushing off, or create a stiff lever to transfer force (3,4). Experimental studies have shown that altering foot properties through custom insoles (5,6) or local anaesthesia of intrinsic muscles (7) had significant effects on ankle-foot and whole-body mechanics and energetics during walking (5–7). Yet, as it is hard to manipulate individual structures (muscles, tendons, ligaments, bones) experimentally, probing their contributions to gait mechanics and energetics has been difficult. Predictive simulations based on a musculoskeletal model provide an alternative approach to study how foot structure shapes whole-body gait. Predictive simulations generate do novo gait patterns based on a musculoskeletal model. Because they do not rely on experimental gait data, changes in the predicted gait can be directly attributed to changes in the model. However, using predictive simulations to study the relationship between foot properties and gait mechanics requires sufficiently detailed foot models. So far, foot models used in predictive simulations were very simple, especially in the light of the complex foot anatomy. Three-dimensional simulations with multi-segment foot models considered up to three segments (talus, hindfoot-midfoot-forefoot, and toes) (8–13), but did not consider a deformable foot arch. Here, we present a novel four-segment (talus, calcaneus, midfoot-metatarsals, toes) musculoskeletal model of the foot that can be used with predictive gait simulations and use this model to study how different foot structures (the longitudinal arch, plantar fascia, plantar fat pads, and muscles actuating the foot) influence simulated whole-body movement.*

2b. Move the supporting information for the foot up (paragraphs that start at L92, L102, L116, L131).

We moved these paragraphs up.

We moved information about the Achilles tendon to its own separate paragraph. This resulted in the windlass mechanism and plantar fat pads also having their own paragraphs.

2c. Discuss predictive simulations with just enough detail as needed to convey its use in the manuscript and to help support the need for a new, but still relatively few (3) segment model.

We have reduced the discussion on predictive simulations in the first paragraph to:

*Predictive simulations generate do novo gait patterns based on a musculoskeletal model. Because they do not rely on experimental gait data, changes in the predicted gait can be directly attributed to changes in the model. However, using predictive simulations to study the relationship between foot properties and gait mechanics requires sufficiently detailed foot models. So far, foot models used in predictive simulations were very simple, especially in the light of the complex foot anatomy. Three-dimensional simulations with multi-segment foot models considered up to three segments (talus, hindfoot-midfoot-forefoot, and toes) (8–13), but did not consider a deformable foot arch.*

The paragraph where we discuss previous predictive simulation studies was moved down, so it comes after the paragraphs about foot mechanics.

*Predictive simulations based on simpler foot models than the one proposed here have already shown that foot structure affects whole-body movement.* *Simplified models of human foot function have been used to explore how foot structure affects whole-body movement. Modelling a compliant foot arch as well as toes in 2D gait simulations increased the cost of transport and more so for lower gait speeds (25). Increasing the stiffness of the longitudinal foot arch resulted in lower cost of transport, both in simulations and experiments (26). But insights based on 2D models are limited given that gait is a 3D movement and that there is considerable out of plane movement at the ankle and foot (e.g. ankle inversion/eversion). In 3D simulations, modelling the toes as a separate segment mainly improved the accuracy of predicted knee kinematics, kinetics, and vasti activity during stance (8). While simulations based on detailed 3D musculoskeletal models already capture many key features of human walking mechanics and energetics, predicting the ankle mechanics remains challenging. Simulations do not capture ankle dorsiflexion during stance (8,12,27), or underestimate ankle plantarflexion in terminal stance (8,10,11,28-30). They capture the peak in soleus activation during late stance, but fail to predict the early onset and slow increase of soleus activity (8,9,11,13,27-31). Reducing the Achilles tendon stiffness (relative to the default Hill-type muscle model (32)) resulted in an earlier onset of soleus activation and a small increase in stance ankle dorsiflexion, yet ankle kinematics remained inaccurate (8). Because foot mechanisms such as the compliant longitudinal arch and the windlass mechanism are thought to support ankle push-off, we expected that using a more detailed foot model would result in more accurate predictions of ankle mechanics.*

3. Introduction: There is limited discussion of previous foot models, and in particular, no discussion of more complicated models. These should be included to give a richer picture of current foot models, and a short discussion should be added as to why these are either not accurate or are not suited for your use. For instance, Maharaj et al., Modelling the complexity of the foot and ankle during human locomotion: the development and validation of a multi-segment foot model using biplanar videoradiography, specifically mentions its possible use in predictive simulations.

We added a paragraph to discuss more detailed musculoskeletal models of the foot, and why we do not use them for predictive simulations as-is. We had included in the methods section that we relied on a more detailed model (Malaquias et al, 2017) to inform some of our modelling choices, but it is a good idea to already include this in the introduction.

*Detailed foot models available in literature are not directly suitable for use in predictive simulation. These models have mainly been developed for use in data-driven analyses, i.e. analyses that used experimental data as input (e.g. marker trajectories, electromyography, or ground reaction forces measured during gait) to infer information about variables that could not be measured (e.g. motion of individual bones, or muscle forces). These multi-segment foot models consider 3 to 26 foot segments, with up to 35 degrees of freedom (33–37). Existing models include up to 30 intrinsic foot muscles and up to 66 ankle-foot ligaments (34,35). Depending on their intended use, the models include inertial properties of the segments, and/or soft tissues (e.g. muscle-tendon units, ligaments). It is not straightforward to use these detailed models for predictive gait simulations that generate de novo movement patterns because only their use for estimating kinematics of individual bones has been validated (33–35). It remains unclear how accurate the estimated muscle and ligament forces are, and such validation is challenging given the high level of detail. We chose to gradually increase the complexity of the foot model starting from the state of the art in predictive simulations and the open questions in foot function outlined above. This incremental approach makes it more tractable to analyse how different modelling choices (e.g. adding a degree of freedom, or selecting a value for a muscle parameter) affect the predicted gait. Our developments were in part informed by a foot model designed for inverse analyses and we believe that such models will continue to inspire models for predictive simulations.*

Maharaj et al. indeed mention that their model can be used for predictive simulations. To my understanding, this statement is based on the ability of the model to capture intrinsic foot kinematics with relatively few degrees of freedom. To use this model in predictive simulations, more soft tissue properties need to be added (foot-ground contact, muscles, ligaments). If we plan to extend our foot model to have an extra degree of freedom, I would definitely try using the joint definitions by Maharaj et al.

4. Introduction: Final paragraph. Since there was limited discussion on previous foot models (including what 2-segment models can’t capture), it’s unclear why at this point a new one is necessary. Further, this makes it more difficult to know what data will be needed to show that the new foot model sufficiently captures salient features that could not be captured before. I believe that this leads to plots with many subpanels, since the focus of the validation is unclear at this point.

We updated the discussion of previous simulation studies to more explicitly state what the limitations of their (foot) models are. We also added a more concrete description of the features of ankle kinematics and soleus activation that they do not capture.

In the final paragraph of the introduction, we added an overview of the different sets of simulations we did.

*Here we present a dynamic four-segment foot model (talus, hindfoot, midfoot-forefoot, and toes) for 3D predictive simulations of gait. The model describes passive stiffness of the midtarsal joint and includes the plantar fascia. The foot is actuated by extrinsic as well as intrinsic foot muscles. We used our model to simulate different conditions. First, we simulated how a standing foot deformed under vertical loading (cfr. experiments (2,18,19)) to evaluate whether our model captured the mechanical properties of the foot. Second, we simulated walking for a healthy adult based on a whole-body model including our new foot model to evaluate how our more realistic foot model influenced whole body kinematics, kinetics, energetics, and muscle activation patterns. We evaluated simulations against experimental data and compared them with state-of-the-art simulations based on a three-segment foot (8). Third, we simulated walking based on a series of foot models with alternative properties to evaluate the effects of modelling choices and to study the contribution of individual foot structures to whole-body gait. Fourth, we simulated experiments that manipulated foot properties during gait. We evaluated how limiting foot arch compression affects the metabolic cost of walking and running (cfr. (5)), and how local anaesthesia of intrinsic foot muscles affects walking kinematics and energetics (cfr. (6)). We identified three properties of the ankle-foot complex that are important to capture ankle and knee kinematics, soleus activation, and ankle power of healthy adults: 1) compliant Achilles tendon, 2) stiff heel pad, 3) the ability to stiffen the foot. The latter requires sufficient arch height and contributions of plantar fascia, intrinsic and extrinsic foot muscles. A reduced ability to stiffen the foot results in walking patterns with reduced push-off power.*

5. For a paper with this much validation, I would have preferred to have the Methods section come before the Results and Discussion sections, if amenable to the journal.
5a. For example, this made it difficult to understand what the “proposed 3-segment foot model” was, when it was unclear what parameter sweeps had been performed and tested, or the differences between the 3-segment model to the two 2-segment models.
5b. L315: “curves from Gene instead of Natali et al.” were used. Unclear why one was chosen over the other if the Methods section comes after.

We moved the materials and methods section to come before the results.

6. The authors should be very careful about comparison claims. They must be clearer about how comparisons were done to help the reader understand the logic, especially since no statistical tests were performed to claim any true differences. While it might be possible to do so since there were 10 gait cycles of data, it would be sufficient to more clearly state how these differences were defined. Some examples include the following:

Statistical tests are typically designed to test for differences. We do know that our model does not capture all details of human musculoskeletal dynamics. Therefore, we thought it was more useful to quantify differences. However, when doing so, we took the standard deviation of the experimental data into account. We added more details on how we interpreted differences between simulations and experimental data or between simulations based on different models to the methods section:

*To evaluate the realism of our simulations, we calculated normalised cross-correlation coefficients between simulated and mean experimental joint angles, moments and powers. We considered the simulated and experimental curves to be in good agreement if the normalised cross-correlation coefficient was above 0.90 (31), and in moderate agreement if it was between 0.70 and 0.90 (cfr. threshold used in (29)). When comparing different simulation results, we used a difference of 0.05 as threshold. We also calculated weighted root mean square errors between simulated and experimental joint angles, moments and powers as*

$$RMSE=\sqrt{\frac{1}{N}\sum_{i=1}^{N} \left( \frac{mean_{i}-simulated_{i}}{{SD}_{i}} \right)^{2}},$$

*where mean_i_ and SD_i_ are the mean and standard deviation of the experimental data for one time point, and simulated_i_ is the simulated value on the corresponding time point (note that simulations were deterministic and therefore had 0 standard deviation). Mesh points of the simulation were chosen as discrete time points (i.e. 200 points per gait cycle). We considered an RMSE below 3 as moderately accurate, and below 2 as accurate (29,31), and set a difference of 1 as threshold for comparisons. We made separate comparisons for stance phase and swing phase.*

6a. For instance, on L214 they claim that subtalar and MTP angles “clearly improved”. However, according to their tables in S10: 1) the subtalar cross-correlation in stance was lower. 2) the MTP cross-correlation in swing was lower. 3) the subtalar weighted RMSE was higher in swing.

There was a mistake in the script we used to calculate the cross-correlation coefficients, which resulted in some values that were conflicting with our description.

We added a description of which features of kinematics our simulations capture, to help readers understand why we consider something as an improvement.

Our interpretation of the difference between simulated and experimental midtarsal kinematics during swing is moved to the discussion section (see response to major comment 7).

*Accuracy of predictive gait simulation with a 4-segment foot model*

*We compared simulated gait patterns using the proposed 4-segment foot model to experimental data to evaluate the realism of our simulations, as well as to simulations based on the 3-segment model we used before (Falisse et al. (8)) (Figure 3, black and orange traces). Normalised cross-correlation coefficients (NCC) and weighted root mean square errors (RMSE) between simulated and experimental gait variables (joint angles, moments and powers) are reported in supplement S11.*

*Using the proposed model improved the overall agreement between simulated and experimental ankle and foot kinematics compared to Falisse’s model. RMSE values decreased and cross-correlation coefficients increased, with exception of the subtalar kinematics during swing (supplement S11). Ankle kinematics showed the largest improvement. We captured the ankle plantarflexion at initial contact, followed by dorsiflexion (Figure 3a). We predicted plantarflexion at the end of push-off, but underestimated its magnitude. We captured subtalar kinematics during stance, except for the inversion at initial contact. We predicted subtalar eversion during swing, but not within the experimentally observed range. We predicted that MTP was extended at initial contact, followed by little motion during most of stance, and a steep increase in extension during late stance. Simulations with Falisse’s model did not capture these features of MTP kinematics. Predicted midtarsal kinematics were accurate for the stance phase (RMSE 1.2, NCC 0.97) but deviated from experimental data for the swing phase. Using the proposed model also led to more accurate stance knee kinematics than using Falisse’s model. The accuracy of the predicted hip flexion increased, while swing hip adduction accuracy decreased.*

*Simulations based on the proposed model resulted in similar ankle, knee and hip moments and ground reaction forces as simulations based on Falisse’s model. The main difference was a decrease in knee moment accuracy (stance RMSE 7.52 vs. 5.13, swing RMSE 19.78 vs. 12.13, NCC changes < 0.05), but a better estimation of the peak knee flexion moment during push-off (Figure b). We also found a slight overestimation of the ankle plantarflexion moment during mid-stance when using the proposed model (Figure b). We could not evaluate the realism of the predicted midtarsal and MTP moments as we did not measure ground reaction forces acting on each foot segment. Simulated ground reaction forces were similar for both foot models (Figure S31). Simulations based on both models predicted the M-shape pattern of the vertical ground reaction forces but displayed a steeper increase after initial contact compared to the measured ground reaction forces.*

6b. On L215, midtarsal kinematics were “very accurate”. How does this differ than just “accurate”? What thresholds were necessary? This could be helped with better information in the introduction that defines what could be a helpful level of accuracy.

We included RMSE and cross-correlation values for midtarsal kinematics during stance, and changed the wording to “accurate” to reflect the thresholds we set (see changes to methods section).

6c. L224: “better estimation of the knee flexion moment during push-off and a slight overestimation of the ankle plantarflexion moment during stance”. The plots are small, so while these trends appear to be correct, it is unclear if these are meaningful differences, or if they would be within noise (especially since stride to stride variability is not described). Instances like this could be helped by reducing the number of subplots, and showing only subplots that are discussed in the main body so that they can be presented larger.

We reduced the number of subplots and made them larger. We specify in the caption that the grey bands reflect the stride to stride variability. Stride to stride variability is also taken into account when computing RMSE (see above).

*Figure 3 Predictive simulation results with the presented model compared to experimental data and state-of-the-art simulation. Vertical lines indicate stance-to-swing transition. (a) Kinematics. (b) Kinetics. (c) Joint powers. (d) Muscle activation. Experimental data was obtained from one subject and therefore the grey band represents stride to stride variability. Kinematics, kinetics, and powers of all joints can be found in supplement S11.*

6d. In general, it was unclear why cross-correlations and RMSEs were calculated but not used as support for many points in the main text. If they aren’t used as support, it may not be necessary to report all of these. Or, if they are important to capture differences and similarities, the main text should discuss and interpret these values.

We focus on qualitative descriptions of gait features (e.g. ankle plantarflexion in late stance , onset timing of soleus activation), because it is hard to capture this kind of observations in numbers but we wanted to provide a more quantitative evaluation as well. We have adjusted the comparisons of the results, to make sure they are consistent with the cross-correlation coefficients and RMSE values (see 6a). We deliberately chose to not include the coefficient values behind each comparison in the main text, because this would make the text difficult to read.

We added a paragraph to the Discussion section where we interpret the normalised cross-correlation coefficients, weighted RMSE values, and qualitative gait features:

*While simulations with our 4-segment foot model resulted in physiologically plausible gait, there were still discrepancies between predicted and experimental gait. Overall, the simulated gait variables showed moderate to good shape-agreement with mean experimental data (cross-correlation coefficients > 0.70). Kinematics and kinetics of most stance leg joints showed good agreement. Most weighted RMSE values were higher than the threshold we set for good agreement (RMSE < 2). Previous simulation studies reported RMSE values below 2, but these were calculated based on data of multiple subjects (29,31). We compared simulation results to data from a single subject and therefore the calculated RMSE values are not directly comparable to those from other studies. Intra-subject variability is smaller than inter-subject variability and therefor our standard deviations were lower, resulting in higher RMSE weights. Simulations with our model did capture many features of ankle-foot kinematics, energetics, and muscle activations (e.g. peak value and timing, onset of increase or decrease) that we observed in our reference data and are reported in the literature. We scaled the model to the anthropometry of the reference subject, but joint definitions, inertial parameters, and mechanical parameters of muscles, tendons, and other soft tissues are still based on an average healthy adult. Personalising the model, such as estimating subject-specific muscle-tendon parameters (79) might improve the agreement between simulated and experimental gait for this subject (i.e. reduce RMSE) but would requires more experimental data. This may not be required to study gait in healthy adults but is important when studying gait pathology for a specific patient.*

7. The authors do a good job of noting shortcomings, but often lack interpretations for how these shortcomings will affect downstream interpretations, which helps to guide readers in which use cases may be good or bad to use this model. Some examples include the following:
7a. For example, L213 “underestimated plantarflexion at the end of push-off”. How does this affect the interpretation of the results or the use of the model by others?

We added to the Discussion section why we expect the differences between simulated and experimental ankle-foot kinematics to have little influence on the interpretation of our results.

*Our simulations did not capture midtarsal and subtalar kinematics during swing and underestimated ankle plantarflexion angle during late stance and swing. Hence, kinematic errors are largest when forces and powers in the ankle-foot complex are low. Therefore, these errors might have little influence when using our model to study the relation between foot structure, and its contributions to load bearing and propulsion.*

To verify that the effect of these kinematic differences was limited to the behaviour of the foot in swing, we used the nominal 4-segment foot model to run a tracking simulation (not included in the manuscript). In this tracking simulation, we added an extra term to the objective function of the optimisation problem. This extra term was the RMSE (non-weighted) between simulated and mean experimental ankle and subtalar kinematics. (Note that this makes the resulting simulation no longer predictive, since it uses gait data). In the results of this tracking simulation, we see that:

1) The model can walk with more ankle plantarflexion without breaking the laws of physics (Figure R1). Tracking had only a limited effect on the predicted kinematics during stance where it was already good.
2) This is less efficient, since each term of objective function is higher (Table R1) (except the joint limit torques term, which is very low either way). However, the increases are relatively low (< 20%).
3) Kinematics of the joints we did not track did not show major differences with the predictive simulation results (Figure R1).

These observations support our idea that the kinematic differences do not invalidate the current study. The results of this tracking simulation provide a starting point for future work to further improve the accuracy of our simulations.

Table R1 Objective function terms

|  | metabolic energy rate | activation | joint accelerations | arm actuators excitation | joint limit torques | tracking |
| --- | --- | --- | --- | --- | --- | --- |
| predictive | 169 | 61 | 75 | 29 | 7.75E-03 | 0 |
| tracking | 179 | 64 | 88 | 31 | 1.35E-03 | 12 |

Figure R1 Tracking mean experimental ankle and subtalar kinematics.

7b. L218: “Using the proposed model also led to slightly different estimates of hip and knee kinematics than using Falisse’s model”. Is this “different” in a good or bad way? Does this affect interpretations? If not, why?

We made “different” more concrete based on the thresholds mentioned above (see 6a for full paragraph):

*Using the proposed model also led to more accurate stance knee kinematics than using Falisse’s model. The accuracy of the predicted hip flexion increased, while swing hip adduction accuracy decreased.*

7c. L285: “2-segment model captured the experimentally observed ankle dorsiflexion and ankle power during the stance phase but failed to capture fine details of foot kinematics”. Which details and why does this matter?

We have made the comparison more concrete, and included why this matters when choosing which model to use.

*To dissociate the effect of modelling an additional foot segment versus altering parameters, we created a new 3-segment model (“nominal 3-segment foot model”). This model has the same complexity as Falisse’s model, with parameters based on our 4-segment model. Simulations based on the nominal 3-segment model captured the experimentally observed ankle dorsiflexion and soleus activation pattern better than simulations with Falisse’s model (Figure 3 dotted lines, supplement S11). Simulations based on the nominal 3-segment model overestimated subtalar range of motion and underestimated MTP flexion at push-off. They also failed to capture peroneus longus activation (Figure S31). The predicted peak ankle power (~2.5 W/kg) is similar to the experimental peak (~2.3 W/kg), but the stance ankle power is less accurate than the power predicted based on the 4-segment foot (RMSE 14.27 vs. 8.53, cross-correlation 0.74 vs. 0.83). Given that the nominal 3-segment foot model captures ankle kinematics well, it could be considered as a simpler alternative for the 4-segment model if the study does not require foot and subtalar kinematics.*

7d. L415: that cost of transport “changed for walking but increased by 10% for running”. 10% is high compared to 6% from the experiment. What were reasons why? Does this invalidate your study or why is this still good for your study?

We initially used a stiffness of 100 Nm/rad for the insole, which was too high. We adjusted the stiffness to better match the described experiment:

*The properties of the insole were chosen such that they reduced arch compression by 80% during level running at 2.7 m/s in agreement with experimental data (6). We simulated gait at 2.7 m/s for different insole stiffnesses, and selected the stiffness (50 Nm/rad) that reduced arch compression by 80% relative to the same speed without insole.*

This resulted in a 4.4% increase in cost of transport for running, and an 0.5% increase for walking.

8. Make sure that there is sufficient information in the main text itself to support points made in the main text, rather than assuming the reader has gone through the supplemental information in whole. For instance, L300: “a sufficiently compliant Achilles tendon…” is not supported by Figure 4, since it is not clear that a large parameter sweep had been performed.

We now explicitly mention that we performed large parameter sweeps in the main text:

*Effects of modelling choices on the predicted gait pattern*

*We evaluated the effect of modelling choices on the predicted walking motion to explore how different foot structures shape walking mechanics and energetics. We compared the effect of a specific modelling choice with respect to the proposed model (“nominal 4-segment foot model”). We discussed only the gait features that are sensitive to the change. The results of additional parameter sweeps are available in the supplementary material. In all figures, black lines represent results based on the nominal model and coloured lines represent results based on a model with alternative parameter values.*

In the subsections about the effect of Achilles tendon stiffness and plantar fascia stiffness, we added a description of the trends we observed from the larger parameter sweep.

*Achilles tendon stiffness*

*Achilles tendon stiffness had a large influence on ankle dorsiflexion during mid-stance. In both the nominal 4-segment and 3-segment foot models, high Achilles tendon stiffness (i.e. normalised tendon stiffness of the generic Hill-type model (34)) resulted in little or no ankle dorsiflexion during midstance in contrast to experimental observations (Figure ). The energy storage in the Achilles tendon and ankle push-off power are lower in the simulations with a stiff Achilles tendon than in the simulations with the lower tendon stiffness that we propose here (i.e. half of the normalised tendon stiffness of the generic Hill-type model (34)). Using a higher versus lower Achilles tendon stiffness also led to a worse agreement between simulated and experimental soleus activity (Figure ). Simulations with different Achilles tendon stiffness values showed that ankle dorsiflexion, ankle power and soleus activation peaks all increased with decreasing stiffness, while mid-stance knee extension decreased (Figure S1).*

*Plantar fascia stiffness*

*Plantar fascia stiffness had a strong influence on ankle push-off. Reducing plantar fascia stiffness (i.e. using stress-strain curves from Gefen (50) instead of Natali et al. (53)) resulted in increased ankle plantarflexion and increased midtarsal extension during stance (Figure a). These kinematics show similarities with pathological gait observed in patients with a midfoot break foot deformity (78). Interestingly, reducing plantar fascia stiffness resulted in lower activation of the intrinsic foot muscle, instead of higher activation to compensate for the reduced stiffness. The muscle fibres underwent an elongation up to 35% of optimal fibre length, which is much larger than in the nominal simulation (Figure d). Sweeping plantar fascia stiffness showed that midtarsal extension decreased with increasing stiffness (Figure S13). Ankle plantarflexion and ankle power increased with plantar fascia stiffness.*

9. It is unclear why the simulations of the custom insoles and of the nerve block only appear in the Discussion section. If it was difficult to decide which info should go into Results or Discussion, it may make more sense to combine the Results and Discussion sections and have subsections for validation and these other experimental tests (if amenable to the journal).

We added a new subsection to the results: “*Simulating experimental manipulations to foot properties*”. We moved the paragraphs about walking and running with restricted arch compression, and walking with local anaesthesia of intrinsic foot muscles there.

10. Make sure that each of the three models is referred to in the same way throughout the manuscript, so that the reader can better follow along. For instance, L568 describes “an alternative 2-segment foot model”, but is this the same as “new 2-segment foot model” in Fig 2? Or the “nominal 2-segment foot model” in L283?

We changed the text and figures to consistently use “nominal 3-segment” and “nominal 4-segment” model.

11. In general, the figures are difficult to read due to their size, and the authors should be careful to make sure that the figures are complete. Some specific examples below:
11a. Figure 1: It’s unclear why the titles are “Foot arch stiffness”, “Midfoot stiffness”, and “Foot arch stiffness”. More detail in the caption and main text would be helpful to guide the reader through this logic.

We updated the titles to be more meaningful:

*Figure 2 Deformation of a standing foot under vertical compression. Full lines indicate experimental data (digitised from (a) Ker et al. (2), (b) Yawar et al. (19), (c) Welte et al. (18)), circles represent simulations. (a) Repeated loading of a cadaver foot after subsequently removing more soft tissue. The table gives an overview of structures that are present in each condition. Elongation was computed as the difference in length between calcaneus origin (defined as the most interior, lateral point on the posterior surface of the calcaneus (74)) and MTP joint centre during the virtual experiment with respect to the resting condition, i.e. the unloaded intact foot. (b) Vertical force-displacement curves for different toe dorsiflexion (DF) positions. Displacement was calculated as the vertical position of the ankle joint centre, relative to its position when no external load is applied to the foot with toes in 0° DF. (c) Vertical loading of a standing foot of an in vivo subject, with toes passively dorsiflexed 30° and -30°.*

11b. Figure 1: Is panel (b) supposed to have a y-axis of BW or kN?

BW, since this is what Yawar et al. used.

11c. Figure 1: It seems like the y-axis and x-axis should be flipped (applying the force seems to be the independent variable, and displacement is the dependent variable).

Force is indeed the independent variable. We chose the axes to be consistent with how the original experimental data was reported.

11d. Figure 3: the blue and green lines are not described in the legends.

The colours are to help relate each line of panel (a) to the associated bar plot in panel (b). We updated the legend:

*Figure 4 Deformation power and work by soft tissue distal to ankle-foot segments. (a) Power distal to segments. (b) Positive, negative, and net work. Experiment-based data, digitised from Takahashi et al.* (70)*, is plotted as reference.*

11e. All figures: the size of the subpanels are too small to see the detail that is noted in the text, often the colors are hard to differentiate, and the use of solid versus dashed lines are unclear. For instance, in Figure 5, a green line over a gray shaded region will not be color-blind friendly. In Figure S15, the color and solid/dashed line choices do not reflect the continuous numbers for the foot-ground contact stiffnesses. If the dashes were intended to help the other lines show up, it is likely better to have bigger subpanels rather than different line types switching back and forth.

We have updated the figures to have larger subplots. We chose to keep the dashed lines as they enable an intuitive comparison between the nominal 4-segment and nominal 3-segment model in figure 5 and dashed lines are colour-blind friendly.

In the main text:

*Figure 5 Effect of Achilles tendon stiffness. A stiff Achilles tendon results in less ankle dorsiflexion and push-off power, and a different soleus activation pattern. Experimental data was obtained from one subject and therefore the grey band represents stride to stride variability.*

*Figure 6 Effect of foot arch stiffness. Fibre length is normalised by optimal fibre length. Experimental data was obtained from one subject and therefore the grey band represents stride to stride variability. We reduced foot arch stiffness, or the ability to stiffen the arch, by removing the plantar intrinsic foot muscle, by reducing plantar fascia stiffness, or by reducing foot arch height. All changes resulted in alterations in kinematics and reductions in peak ankle power.*

In the supplement:

- We have changed page orientation to landscape to better fit the figures.
- We have reduced the unused space between the subplots.
- We have changed line width and the use of dashed lines to improve visibility.

11f. It may be possible to reduce the number of lines on each plot (especially in the supplemental section) by considering the minimal number of lines to convey the point that the authors are trying to make. While the authors may have run every 10% of changes between 30% and 100% (as in Figure S1), fewer lines could be plotted to support the same points, and if important to the authors to provide all of this data, could provide this as data in files rather than as plots.

We have reduced the number of lines in the supplementary figures illustrating the effect of Achilles tendon stiffness, plantar intrinsic muscle optimal force, and arch height.

12. In general, the authors are thorough, but should provide more interpretation for the reader, so that portions of the manuscript are synthesis of previous work (relevant to this manuscript) rather than a list of previous work. Some examples:
12a. For instance, L82-85: Short description of a previous 2D simulation is given, but what was both good about the simulation and what is lacking is not explicit. Providing interpretation and synthesis will better highlight the gap in the current state-of-the-art and support the current work better.

We made it more explicit what the limitations of previous simulation studies were.

*Modelling a compliant foot arch as well as toes in 2D gait simulations increased the cost of transport and more so for lower gait speeds (25). Increasing the stiffness of the longitudinal foot arch resulted in lower cost of transport, both in simulations and experiments (26). But insights based on 2D models are limited given that gait is a 3D movement and that there is considerable out of plane movement at the ankle and foot (e.g. ankle inversion/eversion).*

12b. L398-400: “Interestingly, the energy efficient mechanisms … are the same mechanisms as experimentally observed in running”. A little more commentary here, such as in what ways does this support the confidence and use of this model in new situations, would improve the impact for other researchers.

We extended this section in the discussion.

*Interestingly, our model suggests that the energy efficient mechanisms ... are the same mechanisms as experimentally observed in running. These model predictions should be evaluated experimentally. This example shows how the simulations can be used to generate hypothesis and inspire future experiments.*

13. L642 states that there were 10 gait cycles of data. Were the simulation results throughout the paper an average over these gait cycles? If so, it would be good to be precise on this point.

As our simulations do not account for uncertainty, they only yielded one simulation in every condition (i.e. for every model and imposed speed). We compared simulations to experimental gait data of 10 cycles. We added to the methods section how these data resulted in the mean and standard deviations that we use to compare the simulation results to:

*We compared simulation outcomes to previously published experimental data (8). As our simulations do not take into account uncertainty, they yielded one solution per condition (i.e. combination of model parameters and imposed gait speed) whereas we used experimental data from 10 gait cycles. The data (marker trajectories, ground reaction forces, surface electromyography) was collected from a healthy female adult (mass: 62 kg, height: 1.70 m, age: 35) during 10 gait cycles of overground walking at self-selected speed (1.33 m/s) (8). The subject was instrumented with 59 retro-reflective skin-mounted markers. There were 10 markers mounted on each foot (supplement S10). Additionally, we measured (with measuring tape) the normal distance from the medial malleolus centre and the first metatarsal head centre to the ground while the subject was in a seated position with the foot flat on the ground. We scaled a musculoskeletal model to the anthropometry of the subject, as described in the section on the musculoskeletal model. We performed inverse kinematic and inverse dynamic analysis (38) based on the model with 4-segment feet and selected midtarsal joint orientation (Figure 1), and calculated joint powers for each individual gait cycle. After time-normalising the processed data, we calculated mean and standard deviation at 100 points equally spread over the gait cycle duration. For the inverse dynamic analysis, we applied experimental ground reaction forces on the calcaneus, thus we did not obtain moments nor powers for the midtarsal and MTP joints. Foot joint moments are sometimes estimated by applying the total ground reaction forces to a more distal segment once the centre of pressure moves anterior to the joint (71). This method assumes that only one foot segment is in contact with the ground at each instance in time. We did not make this assumption in our model and therefore we did not compare simulated moments to moments computed based on experimental data.*

14. A description of the simulations of the experiments with insoles and the nerve block were not included in the Methods section.

We added a description to the methods section. We added a how we determined the stiffness of the insole (see comment 7d).

*To test whether simulations with our model can capture the effects of manipulating foot properties, we simulated walking and running with an insole that limits arch compression (6) and walking with local anaesthesia of intrinsic foot muscles (7). To model the insole, we added a spring to the midtarsal joint, that only engages when midtarsal joint angles are positive. The properties of the insole were chosen such that they reduced arch compression by 80% during level running at 2.7 m/s in agreement with experimental data (6). We simulated gait at 2.7 m/s for different insole stiffnesses, and selected the stiffness (50 Nm/rad) that reduced arch compression by 80% relative to the same speed without insole. We then simulated walking at 1.33 m/s with this insole. To mimic the effect of a nerve block inhibiting intrinsic foot muscle activation, we modelled that the activation should remain equal to its lower bound (0.05 cfr. (11)).*

**Minor comments:**

1. It would be helpful if the supplemental material was arranged in a way that, when cited, increased in strictly numerical order along with the main text. It was difficult to find certain pages. This may also help if the supplemental material had page numbers on them that aligned with the table of contents.
2. We have added page numbers, and arranged the supplement in the order that the material is first cited in the main text.

2. L76-L78: I cannot follow along with this topic sentence. Perhaps there is a word missing?

This sentence got removed when updating the introduction.

3. L278: Could change “paragraphs” to “sections”, so that the following sections could be broken down into separate paragraphs. In particular, this could help the section “Plantar fascia and intrinsic foot model” since the different pieces of the model that were studied could be separated into separate paragraphs.

We split this section into separate sections for plantar fascia and intrinsic foot muscle.

4. L341: A definition for the acronym IM is given, but not what the acronym “IM” stands for. Perhaps a symbol like “\tilde{l}^m” could be used?

We changed it to “Fibre length (-)”, and included in the caption that the fibre length is normalised by optimal fibre length.

5. L374-375: Why do the simulations “confirm” in one case and “suggest” in another? It was unclear why the support for one of these statements should be stronger than the other.

We have included a more concrete description of what we see, instead of writing “confirm”.

*Our simulations showed that a stiff foot is required to efficiently generate high ankle powers. It has been suggested that the ability to stiffen the foot is important to generate a powerful push-off (24). In simulation, we could easily manipulate the ability to stiffen the foot by altering arch height, plantar fascia stiffness, or the properties of intrinsic foot muscles. When we reduced the ability to stiffen the foot, simulated peak push-off ankle power decreased (Figure 5b). The magnitude and timing of negative ankle power (i.e. absorbing energy) were different for each alteration. All three changes caused the energy stored in the Achilles tendon to be approximately 18% lower than in simulations with the nominal 4-segment foot model. Our simulations suggest that if the foot cannot act as a sufficiently stiff lever, an optimal strategy is to walk with a flexed knee. This is in agreement with the hypothesised contribution of increased foot compliance to crouch gait in children with cerebral palsy (80). An alternative strategy is to walk with excessive midtarsal extension, and to rely on elastic energy storage in plantar fascia and plantar ligaments (Figure S34). It is important to note that this midtarsal extension is far outside the range of motion of the data from which the functional axis was determined, thus the axis orientation might be different in a person with midfoot break. Nevertheless, these results highlight the potential of the presented model to study the relation between foot pathology and common pathological gait patterns (i.e. midfoot break (78) and crouch gait (81)).*

6. L391: It might be better to soften language that measuring certain quantities is “impossible”. A possible change is to say that it just “has not been done experimentally”.

We changed this in the manuscript.

7. L495: While it may be clear that the curve from Gefen was too compliant, Natali looks a little bit stiffer than measurements too. Was a model between those two tested?

We added the data from Wright and Rennels (1964, 4 specimen) to Figure S12. The parameters reported by Natali et al. were fitted based on this data.

 *Figure S12 Plantar fascia stress-strain. Experimental data digitised from Kitaoka et al. (17), Fessel at al. (18), and Wright and Rennels (19). Since Kitaoka et al. report force, and no cross-sectional area, we calculated the stress assuming a cross-sectional area of 42 mm². This value is based on measurements of cadavers of similar ages (17,18,20). Plantar fascia models taken from Natali et al. (21), and Gefen (12).*

We did not test other stress-strain characteristics but we did test different cross-sectional areas. We added a description of these results to the results section (see major comment 8).

With a previous iteration of the model, we did test other stress-strain characteristics (Figure R2). We decided to not include the plantar fascia based on Song et al. (2011), because it resulted in similar gait patterns as with the plantar fascia model from Gefen (2002). Simulations with a plantar fascia described by a Young’s modulus of 350 MPa (based on finite element studies), resulted in gait patterns that were comparable to the ones obtained with the model from Natali et al.

Figure R2 Plantar fascia stress-strain

8. Closely review the text for grammar. For instance, L506 uses the present tense while much of the paragraph is written in the past tense.

We closely reviewed the text and hope that it is free of grammar errors now.

9. Not all supplemental figures are referred to in the main text. The authors may want to consider removing some of the supplemental figures for brevity, and/or moving them to files online rather than printed as plots.

We included reference to the supplement on orientation of the midtarsal and MTP joint axes in the methods section:

*We modelled the foot arch as a midtarsal joint with a single rotational degree of freedom, connecting calcaneus and midfoot segments. Midfoot and forefoot segments are rigidly connected. Midtarsal joint centre, segment definitions, and segment mass properties were taken from Malaquias et al. (37). The orientation of the midtarsal joint axis is set according to the mean finite helical axis calculated from the motion capture data during the stance phase of walking using the algorithm provided by Ancillao (49)(See supplement S2 for sensitivity of midtarsal joint axis orientation). We defined the rotational stiffness of the midtarsal joint by combining the contributions of long and short plantar, and spring ligaments (37), considering a nonlinear stress-strain characteristic (50) (supplement S3).*

We removed figures that were not referenced.

We prefer plots bundled in the supplementary, so readers can easily access them.

10. For the weighted RMSE, how was the weight computed? Was there a different weight per time point? And if so, how many time points were used for the discretization? It may be helpful to include an equation to show how this was done, especially to make interpretation of the RMSE values in the supplemental figure easier to interpret (e.g., are these unitless or not?).

We added an explanation and equation to the methods section:

*We also calculated weighted root mean square errors between simulated and experimental joint angles, moments and powers as*

$$RMSE=\sqrt{\frac{1}{N}\sum_{i=1}^{N} \left( \frac{mean_{i}-simulated_{i}}{{SD}_{i}} \right)^{2}},$$

*where mean_i_ and SD_i_ are the mean and standard deviation of the experimental data for one time point, and simulated_i_ is the simulated value on the corresponding time point (note that simulations were deterministic and therefore had 0 standard deviation). Mesh points of the simulation were chosen as discrete time points (i.e. 200 points per gait cycle).*

**Reviewer 2:**

**Major comments:**

1. One of the major conclusions of this study is that ‘the ability to stiffen the foot’ is important for recreating the walking mechanics of healthy adults. Within the modeling framework, this ‘ability to stiffen the foot’ is seemingly related to 1) reducing the plantar fascia stiffness, 2) reducing arch height, and 3) removing the intrinsic muscle. While the first two affected the midtarsal kinematics consistent with a less stiff midtarsal joint, it appears that removing the intrinsic muscles had no effect on the midtarsal kinematics (Figure 5). Lines 323-324 also mention that there was little change in the midtarsal moment. Thus, there is no objective support to claim that removing intrinsic muscles led to a less stiff foot (or midtarsal). Can the authors clarify how their data would support the claim that the intrinsic foot muscles affect the ability to stiffen the foot? This is important, considering the main conclusions of this manuscript.

Thank you for this comment. We see how this might have been confusing. Removing the intrinsic foot muscle had only a small effect on the stiffness (relation between joint moments and angles) of the foot as compared to the plantar fascia and foot arch height. Yet, this effect might be meaningful because even though other structures including the extrinsic foot muscles might be able to compensate for the absence of the intrinsic foot muscles to keep the stiffness high, the effect of removing the intrinsic foot muscle on ankle and especially knee kinematics was high. In particular, the reduced knee extension at push-off is not in agreement with experimental data. We have updated the text to explain this and we have added an extra figure to clarify how extrinsic foot muscles, and a difference in operating length of the plantar fascia, are used to compensate for the absence of the intrinsic foot muscle.

*Removing intrinsic foot muscles mainly affected the stance knee kinematics and ankle push-off power, possibly because extrinsic foot muscles that also span the ankle partially compensated for the absence of the intrinsic foot muscles to keep foot stiffness high. Removing the plantar intrinsic muscle resulted in increased stance knee flexion and earlier ankle dorsiflexion, which were not in agreement with experimental data (Figure a). Midtarsal and MTP kinematics remained largely similar. Ankle, midtarsal, and MTP moments were higher in mid-stance, and had a lower peak in terminal stance (Figure 7a). The subtle changes in midtarsal and MTP kinematics resulted in an increased plantar fascia strain and hence a slightly higher plantar fascia moment around both joints (Figure b). During mid and late stance, the midtarsal flexion moment (i.e. resisting arch compression) by extrinsic foot muscles increased. The increased contribution of the extrinsic foot muscles was also reflected in the increased ankle plantarflexion moment in mid-stance (Figure b). However, extrinsic muscles and plantar fascia did not fully compensate for the contributions of the intrinsic muscle to the peak midtarsal and MTP moments in the nominal simulation, and hence the net effect of removing the intrinsic foot muscles was a reduced stiffness of the foot at push off. Removing the plantar intrinsic muscle resulted in lower peak triceps surae activation (Figure S35) and a lower peak ankle moment at push-off (Figure ). The reduction in triceps surae knee moment, combined with increased quadriceps and decreased hamstrings knee moments, resulted in a lower knee flexion moment in mid-stance, which was not in agreement with experimental results (Figure a)Figure . Remarkably, the simulated gait was not sensitive to parameters of the plantar intrinsic muscle. The maximal isometric force needed to be changed to at least double or half its nominal value for changes in gait kinematics to occur (Figure S14). The optimal fibre length and tendon slack length of the plantar intrinsic muscle only influenced its normalised fibre length (Figure S15, S16).*

*Figure 6 Effect of foot arch stiffness. Fibre length is normalised by optimal fibre length. Experimental data was obtained from one subject and therefore the grey band represents stride to stride variability. We reduced foot arch stiffness, or the ability to stiffen the arch, by removing the plantar intrinsic foot muscle, by reducing plantar fascia stiffness, or by reducing foot arch height. All changes resulted in alterations in kinematics and reductions in peak ankle power.*

*Figure 7 Effect of removing the plantar intrinsic muscle on joint moments. (a) Total joint moment. Experimental data was obtained from one subject and therefore the grey band represents stride to stride variability. (b) Joint moment by different muscle groups and plantar fascia. Extrinsic muscles crossing MTP are flexor digitorum and hallucis longus and extensor digitorum and hallucis longus. Extrinsic muscles crossing MTJ (midtarsal joint) additionally include tibialis anterior and posterior, and peroneus (brevis, longus, and tertius). Joint moments by individual muscles are shown in Figure S36.*

2. Line 642: it indicates that experimental data came from a single subject. Yet, the experimental data show standard deviations. Are the standard deviations the variance across the gait cycles from a single subject? Please clarify.

Yes. We added more explanation on how we processed the experimental data to obtain the used reference data:

*We compared simulation outcomes to previously published experimental data (15). The data (marker trajectories, ground reaction forces, surface electromyography) was collected from a healthy female adult (mass: 62 kg, height: 1.70 m, age: 35) during 10 gait cycles of overground walking at self-selected speed (1.33 m/s) (15). The subject was instrumented with 59 retro-reflective skin-mounted markers. There were 10 markers mounted on each foot (supplement S10). Additionally, we measured (with measuring tape) the normal distance from the medial malleolus centre and the first metatarsal head centre to the ground while the subject was in a seated position with the foot flat on the ground. We scaled a musculoskeletal model to the anthropometry of the subject, as described in the section on the musculoskeletal model. We performed inverse kinematic and inverse dynamic analysis (38) based on the model with 4-segment feet and selected midtarsal joint orientation (Figure 1), and calculated joint powers for each individual gait cycle. After time-normalising the processed data, we calculated mean and standard deviation at 100 points equally spread over the gait cycle duration. For the inverse dynamic analysis, we applied experimental ground reaction forces on the calcaneus, thus we did not obtain moments nor powers for the midtarsal and MTP joints. Foot joint moments are sometimes estimated by applying the total ground reaction forces to a more distal segment once the centre of pressure moves anterior to the joint (71). This method assumes that only one foot segment is in contact with the ground at each instance in time. We did not make this assumption in our model and therefore we did not compare simulated moments to moments computed based on experimental data.*

We also made this explicit in the captions of figure 3,5,6,7:

*Experimental data was obtained from one subject and therefore the grey band represents stride to stride variability.*
