# Supplementary material for: A dynamic foot model for predictive simulations of human gait reveals causal relations between foot structure and whole-body mechanics
Source: PLoS Comput Biol. 2024 Jun 20;20(6):e1012219. doi: 10.1371/journal.pcbi.1012219 (PMC11218950; doi:10.1371/journal.pcbi.1012219)
Supplement: Attachment — Submitted filename: DHondt_2024_rev_response_to_reviewer.docx [file pcbi.1012219.s003.docx]

**Response to reviewers**

**General comments**

We thank the reviewers for their thorough reviews and constructive feedback.

Based on comments 4-6, we have used the Grammarly writing tool to find and correct grammatical mistakes. We also had colleagues whose 1^st^ language is English proofread the manuscript and we improved grammar and wording based on their feedback.

We added sub-headers to the results section “Accuracy of predictive gait simulations with a 4-segment foot model” to separate the kinematics, moments, etc.

We formatted figures and captions based on the journal guidelines.

In this document, reviewer comments are in blue. Text from the manuscript is *italic* with additions and changes *underlined*.

**Reviewer 1:**

**Comments:**

1. How did the time to simulate the walking motions different between the different foot models? Providing this information in a sentence or two would help support that your proposed model is indeed just as tractable to use as typical musculoskeletal models with simpler feet.

Simulation time could indeed influence the choice of a model. We added information about the simulation time in the results section, in the subsection where we describe the effects of the number of foot segments.

*(…) Given that the nominal 3-segment foot model captures ankle kinematics well, it could be considered a simpler alternative for the 4-segment model if a study does not require foot and subtalar kinematics. The number of foot segments had little effect on simulation time. The gait simulation converged in 3 hours and 50 minutes when using the nominal 3-segment foot model and in 4 hours and 12 minutes when using the nominal 4-segment model. Simulations with alternative model parameters converged within 2-6 hours.*

Since simulation time depends on the computer, we included the relevant specifications of the computer we used in the methods section.

*Based on convergence analysis (supplement S9) we selected 100 mesh intervals, cold-start initial guess and convergence tolerance of 1e-04. All results shown were obtained from simulations with these settings. To evaluate the sensitivity of predicted gait to differences in the musculoskeletal model, we performed simulations for a range of models and model parameters. All simulations were run on a laptop (allocating two threads of an intel core i7-11850 CPU to each simulation).*

2. Currently, paragraphs, especially in the introduction section, feel more like a list of literature that was reviewed rather than a synthesis of main points. Combining sentences would improve flow and readability, and help readers better understand the logic of each paragraph. A couple examples are provided below (though there are many other opportunities too):
- L97-L99: While the body moves over the standing foot, energy is stored in the Achilles tendon which rapidly releases energy to generate a powerful forefoot push-off.
- L118-121: The intrinsic muscles in the superficial layers of the plantar aspect of the foot have short fibres, a high pennation angle and a long tendon, which enables these muscles to produce force to tension their tendon to absorb and return elastic energy in a controlled way.

We adapted several sentences in the introduction section to improve the flow and readability.

*During walking, most push-off power comes from the triceps surae and Achilles tendon (14). While the body moves over the standing foot, energy is stored in the Achilles tendon which rapidly releases ~~Releasing~~ the stored energy to generate~~s~~ a powerful forefoot push-off (1,14). ~~This power is mainly attributed to rapid energy release from the Achilles tendon (14). Due to its twisted morphology, the Achilles tendon can achieve larger strains than other tendons (15). An efficient push-off requires the foot to act as a stiff lever, such that energy dissipation in the foot is minimal (16,17).~~ This process is facilitated by the twisted morphology of the Achilles tendon, enabling it to endure large strains and store a substantial amount of energy compared to other tendons (15). To use this energy effectively during push-off, the foot must function as a stiff lever to minimise energy dissipation (16,17). Hence, foot stiffness influences the propulsive power generated around the ankle (5,7). Therefore, we investigated how Achilles tendon stiffness influenced simulated gait mechanics using a foot model with compliant longitudinal arch.*

*Intrinsic foot muscles can modulate foot stiffness and might therefore play an important role in optimising efficiency by enabling the foot to function both as an elastic structure that stores and releases energy, and as a stiff lever. The intrinsic muscles in the superficial layers of the plantar aspect of the foot have short fibres, a high pennation angle and a long tendon (20,21)~~. Muscles with similar architecture are suited~~ , which enables these muscles to produce force to tension their tendon to absorb and return elastic energy in a controlled way (22). Multiple plantar intrinsic muscles originate on the calcaneus and insert on different toe bones (20,21). ~~Contraction of~~ Thus contracting these muscles will add to the stiffness of the foot in parallel with the stiffness provided by the plantar fascia (4). Electromyographic recordings ~~showed~~ have demonstrated that intrinsic foot muscles are active during the stance phase of walking (4,23,24). This activity is highest in the second half of the stance phase, when the triceps surae contract and the ankle plantarflexion moment is high (4,23). Preventing intrinsic muscle contraction by injecting a nerve block decreased push-off power of the ankle-foot complex, yet arch deformation and cost of transport were unaffected (7). Modelling intrinsic foot muscles allowed us to elicit their contribution to foot stiffness and walking mechanics.*

3. L713-715: It’s interesting that such a small change in midtarsal extension led to an 18% increase in force. However, it is difficult from the figure cited (Figure S39) to see that there’s much of a change. Can you provide a numerical value in text to make it clearer for readers?

We included the peak plantar fascia strain from both simulations.

*(…) During late stance, our simulations showed an increase in midtarsal extension due to the nerve block (Figure S39). The corresponding increase in plantar fascia strain (3.75% instead of 3.34%) resulted in an 18% increase in force, which compensated for the reduction in force produced by the intrinsic muscle. In both cases the summed force of plantar fascia and intrinsic muscle had a peak value of two times body weight. (…)*

4. Continue to review the manuscript for typographical issues. Here are some that were noted during this current review:
- L58: transfers -> transfer
- L70: do novo -> de novo
- L176: (add a comma) … designed for inverse analyses, and we believe that…
- L225: with -> by
- L254: (remove a comma) … as a single Hill-type muscle because they act…
- L307: reference issue
- L381 equation: RMSE, mean, simulated, and SD should not be italicized
- L430: we calculated mean -> we calculated the mean
- L537: Figure 3e does not exist.
- L754: Perhaps “weights -> values”?
- L770: remove the comma
- Add spaces between references to literature and figures (e.g., L222, L227, L235, L710)
- Figure S31 caption: There is a comma splice in the sentence describing the two heads of the gastrocnemius.

We have corrected these mistakes.

5. Continue to make sure that verb tenses are consistent with the context of the sentence. Some instances include the following:
- L74: were -> have been
- L76: considered -> have considered
- L77: did not consider -> have not considered
- L577: discussed -> discuss

We have corrected these mistakes.

6. Continue to review phrases in relation to comparisons being made to remove adjectives/adverbs that are not well-defined and do not help support a point or conclusion. Some examples include the following:
- L47: remove “easily”. This seems more subjective to me, and the more important point is that your work now enables other researchers (regardless of how easy it may or may not be for other researchers).
- L186: consider removing “more realistic”. The key point here is that you are validating your model.
- L531: avoid claiming “clearly a better prediction” (and in particular, the word “clearly”). Perhaps one way to reword: “The peak ankle power simulated based on the four-segment foot model (~2 W/kg) better predicted the experimental peak ankle power (~2.3 W/kg) than Falisse’s model (~1 W/kg).
- L601: remove “slightly”
- L774: remove “easily”
- L797: Consider changing “However, in simulations, this information is readily available” to “However, simulations enables us to estimate these contributions”.

We removed subjective adjectives and adverbs from comparisons.

We changed the wording on L797:

*However, in simulations, ~~this information is readily available.~~ we can calculate the exact contribution of each individual element to (the energetics of) the predicted gait pattern.*

I would reserve “estimate” for when we use a model to analyse experimental data. Then we use the model to estimate a variable that we did not measure. In predictive simulations, the variable is exact for the simulated movement. So here I would not consider it an estimate.
